# De novo assembled salivary gland transcriptome and expression pattern analyses for *Rhipicephalus evertsi evertsi* Neuman, 1897 male and female ticks

**DOI:** 10.1038/s41598-020-80454-3

**Published:** 2021-01-15

**Authors:** Ronel Pienaar, Daniel G. de Klerk, Minique H. de Castro, Jonathan Featherston, Ben J. Mans

**Affiliations:** 1 Epidemiology, Parasites and Vectors, Agricultural Research Council-Onderstepoort Veterinary Research, Onderstepoort, Pretoria, South Africa; 2 Department of Veterinary Tropical Diseases, University of Pretoria, Pretoria, South Africa; 3 Agricultural Research Council-Biotechnology Platform, Onderstepoort, Pretoria, South Africa; 4 Department of Life and Consumer Sciences, University of South Africa, Johannesburg, South Africa

**Keywords:** Entomology, Computational biology and bioinformatics, Parasitic infection

## Abstract

Ticks secrete proteins in their saliva that change over the course of feeding to modulate the host inflammation, immune responses, haemostasis or may cause paralysis. RNA next generation sequencing technologies can reveal the complex dynamics of tick salivary glands as generated from various tick life stages and/or males and females. The current study represents 15,115 Illumina sequenced contigs of the salivary gland transcriptome from male and female *Rhipicephalus evertsi evertsi* ticks of early, mid and late feeding stages from 1320 separate assemblies using three short read assemblers. The housekeeping functional class contributed to the majority of the composition of the transcriptome (80%) but with lower expression (51%), while the secretory protein functional class represented only 14% of the transcriptome but 46% of the total coverage. Six percent had an unknown status contributing 3% of the overall expression in the salivary glands. Platelet aggregation inhibitors, blood clotting inhibitors and immune-modulators orthologous to the ancestral tick lineages were confirmed in the transcriptome and their differential expression during feeding in both genders observed. This transcriptome contributes data of importance to salivary gland biology and blood feeding physiology of non-model organisms.

## Introduction

The Ixodidae share the most significant common denominator of blood feeding behaviour with all other blood-feeding arthropods: the host-vector interface. Hosts on which ticks feed possess effective defence mechanisms to prevent blood loss and possible infection. Tick saliva in turn forms the key to successful feeding by counteracting the hosts’ wound healing responses and innate and acquired immune responses during prolonged feeding^1^. This is due to the expansion of certain dominantly expressed protein families in their saliva^2,3^. In this regard, an understanding of the biology of tick feeding, the hosts’ immune evasion tactics and genes involved in these processes contribute to the ongoing search towards improved tick control methods.

Transcriptomics enabled by RNA next generation sequencing technologies (RNA-Seq) has revealed the complex dynamics of the salivary glands of ticks as evident in the growing list of published tick salivary gland transcriptomes generated from various tick life stages and/or genders^4^. These studies identified thousands of transcripts coding for numerous protein families secreted over the course of feeding with defined differential expression patterns between male and female ticks. This approach shaped the concept of ‘sialome switching’, which is hypothesised to aid the tick in feeding for days on the host and still evade the host’s immune system^5–7^. Transcriptomes enabled identification of orthologs and paralogs i.e. genes that started diverging by speciation or duplication, that allow tracing of protein functionality to common ancestors. Transcriptomics also allow for the cataloguing of known and unique genes that can be used to identify proteins using proteomic methods. This is especially relevant for neglected, emerging and re-emerging diseases, such as tick toxicosis and tick induced paralysis, caused by non-model organisms^8^.

*Rhipicephalus evertsi evertsi* is an economically important two-host tick widely distributed across sub-Saharan Africa. It is the major trans-stadial vector of *Theileria equi* and *Babesia caballi*, making it an important agent of equine piroplasmosis^9^. Adults prefer large mammals, especially cattle and Equidae. However, the synchronised appearance of adults in large numbers during spring in certain areas in South Africa, burden lambs extensively and cause an ascending paralysis^10,11^ comparable to the phenotypic paralyses caused by other ticks^12^. To date, no molecular data exist that can explain the ability of *R. evertsi evertsi* to modulate inflammation, host immune responses, haemostasis or cause paralysis. We therefore sequenced the salivary gland transcriptome of male and female *R. evertsi evertsi*, over the course of feeding. The aim of this study was to de novo assemble a gene catalogue of *R. evertsi evertsi* salivary gland proteins of early, mid and late feeding stages. This enabled the identification of genes putatively involved in feeding as extrapolated from expression profiles of protein families during the different feeding stages of both sexes. The catalog may also be used in future as resource for identification of functional proteins.

## Results

### Transcriptome assembly and completeness

**Figure 1:**
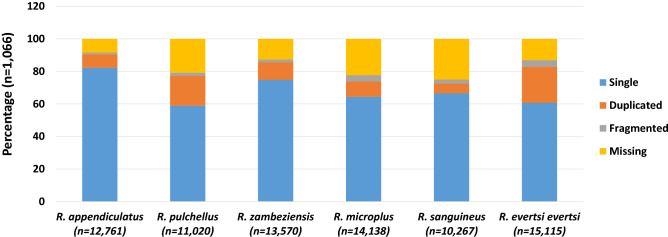
Comparison of BUSCO values of *Rhipicephalus evertsi evertsi* and transcriptomes published from other *Rhipicephalus* species. Indicated in parenthesis is the number of transcripts from each transcriptome submitted to BUSCO analysis. The percentage of single, duplicated, fragmented and missing genes are presented as a percentage fraction contributing to the whole transcriptome. The number of ORFs analysed are indicated in parenthesis.

Sixty-six datasets were generated from different feeding stages for male and female salivary glands. Rigorous adapter and quality trimming removed 44–58% of the reads summarized in Table S1. Using various assemblers and kmer sizes, the datasets were used to de novo assemble the different time points of the male and female salivary glands of *R. evertsi evertsi* yielding 1320 separate assemblies. Open reading frames (ORFs) were extracted from the assembled contigs and clustered at 95% identity using CD-HIT^13^. The ORF set was reduced by selection of coverage cut-offs (reads per million, RPKM > 5; transcripts per million, TPM > 1) and a BLASTX cut-off of E-004. All ORFs (TPM ≥ 1) were manually inspected for duplicate sequences by BLASTP^14^ analysis and alignment before selecting those with the highest percent identity, bit score and complete domains to reduce redundancy to a minimum dataset (MDS) of 15,115. The annotation completeness and transcriptome assembly quality as determined by the Benchmarking Universal Single-Copy Orthologs (BUSCO)^15^ reference-based metrics graded the transcriptome as 82.9% complete with 60.7% as complete single genes, 22.2% as complete duplicated genes, 4.0% fragmented and 13.1% missing. These values compare well with those from other *Rhipicephalus* transcriptomes (Fig. 1).

### Comparison of the *R. evertsi evertsi* assembled transcriptome to transcriptomes of ticks from the *Rhipicephalus* genus

**Figure 2:**
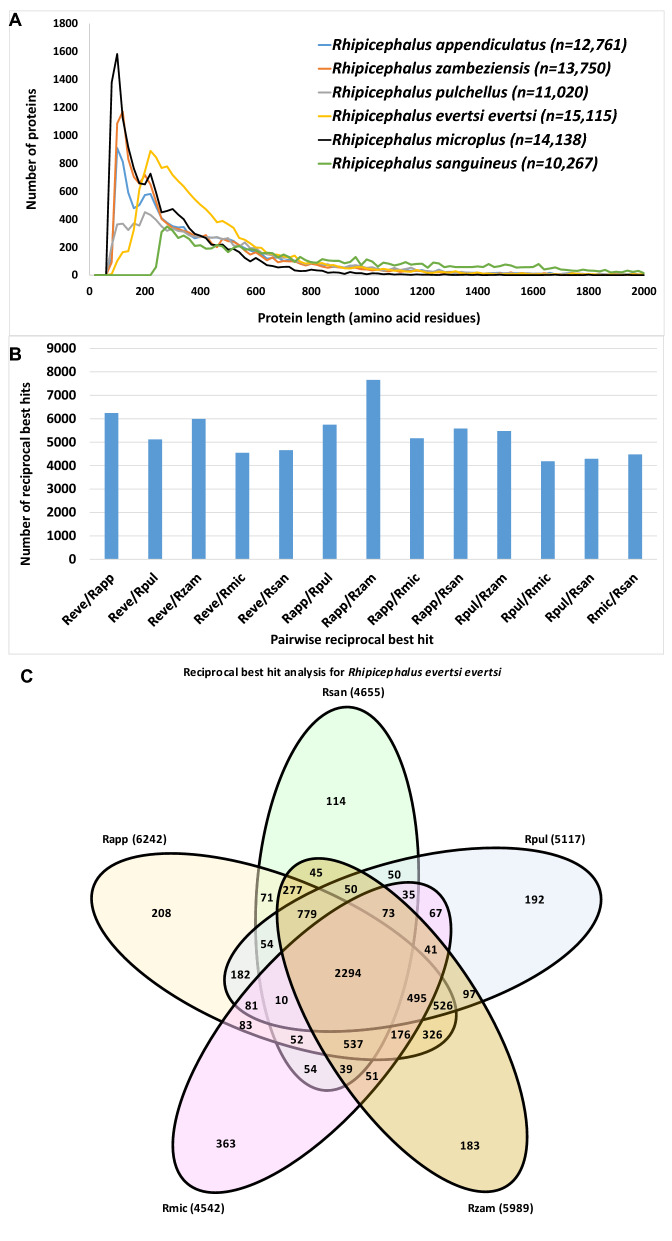
Comparison of the *Rhipicephalus evertsi evertsi* (Reve) transcriptome with other publically available *Rhipicephalus* transcriptomes that include *Rhipicephalus appendiculatus* (Rapp), *Rhipicephalus microplus* (Rmic), *Rhipicephalus pulchellus* (Rpul), *Rhipicephalus sanguineus* (Rsan) and *Rhipicephalus zambeziensis* (Rzam). (**A**) A plot of the protein length in amino acid residues presented up to 2000 residues against the number of proteins. Proteins were binned in windows of 20 based on protein length. (**B**) Pairwise reciprocal best hit analysis for the publically available *Rhipicephalus* transcriptomes. (**C**) Venn diagram to show the number of shared orthologs found for the *Rhipicephalus evertsi evertsi* transcriptome based on reciprocal best hits. Similar diagrams can be found for the other publically available *Rhipicephalus* transcriptomes in the supplementary material. Numbers in parenthesis indicate total number of reciprocal best hits for each transcriptome and while those shared among transcriptomes are indicated in numbers.

To obtain a comprehensible assessment of the quality and completeness of the transcriptome, the 15,115 ORFs were translated to proteins and compared with proteins from publicly available *Rhipicephalus* transcriptomes. To get an estimate on whether the proteins from *R. evertsi evertsi* are shorter than other *Rhipicephalus* transcriptomes (indicating truncated transcripts), the protein lengths from various transcriptomes were compared (Fig. 2A). This confirmed that the translated proteins from *R. evertsi evertsi* are generally longer than the other *Rhipicephalus* transcriptomes, except for *Rhipicephalus sanguineus*. For an estimate of shared orthologs, the transcriptome was compared with the transcriptomes of *Rhipicephalus appendiculatus*^16^*, Rhipicephalus microplus*^17,18^, *Rhipicephalus pulchellus*^19^, *R. sanguineus*^20^ and *Rhipicephalus zambeziensis*^21^ using the reciprocal best hit approach (RBH). Pairwise comparisons indicated that RBHs between transcriptomes are similar, suggesting that the *R. evertsi evertsi* transcriptome possessed similar numbers of orthologs when compared to existing transcriptomes (Fig. 2B). A Venn diagram plot indicated that *R. evertsi evertsi* shared 7726 orthologs in total with all *Rhipicephalus* transcriptomes analysed (Fig. 2C). A total of 2294 orthologs was shared between all transcriptomes, while 5432 orthologs were shared by two or more transcriptomes. Similar results were obtained for the other *Rhipicephalus* transcriptomes (supplementary material), indicating that the *R. evertsi evertsi* transcriptome is comparable to these published transcriptomes. The total number of orthologs could be classified as 83% housekeeping and 12% secretory (classification below).

### Annotation and predicted protein classes

The transcriptome was annotated with sequence similarity searches to proteins in at least one of the listed databases. Six percent (n = 869) of the ORFs could not be annotated with a known function but contributed 3% of the expression represented by TPM values with a dynamic transcript expression range from TPM 1 to 3321. This underlined the fact that many expressed proteins with potential physiological and biological functions, remains uncharacterized. Fourteen percent (n = 2040) could be annotated with a putative secretory protein function contributing 46% of the expression while the majority (80%, n = 12,206) represented a housekeeping function contributing 51% to the overall expression.

### Expression profiling and transcript abundance by functional class in *R. evertsi evertsi* salivary glands

The *R. evertsi evertsi* transcriptome displayed a range of expression over time in terms of transcripts per million (TPM) from the lowest expressed (TPM = 1) to the most abundant (TPM = 13,234). A moderate positive Pearson’s correlation was obtained over the whole transcriptome between expression and abundance (counts) of transcripts of each class (*r*(85) = 0.5083, *p* < 0.05) as well as between the most abundant transcripts and their expression (*r*(20) = 0.7096, *p* < 0.05). On the other hand, the highly expressed transcripts were not necessarily the most abundant (*r*(20) = 0.331, *p* < 0.05) with the twenty most abundant proteins contributing ~ 57% to the total expression based on TPM values. The majority of these were housekeeping proteins involved in metabolism, genetic information processing, cellular processes and organismal systems. Secretory proteins also highly expressed were the glycine-rich family (26%), lipocalins (5%), trypsin inhibitor-like cysteine rich (TIL) domain proteins (3%), immunoglobulin-binding proteins (2%), Kunitz-type inhibitors (BPTI) (2%), the 8.9 kDa protein family and metalloproteases, each contributing ~ 1% to the total expression in the transcriptome respectively.

#### Housekeeping functional class

**Figure 3:**
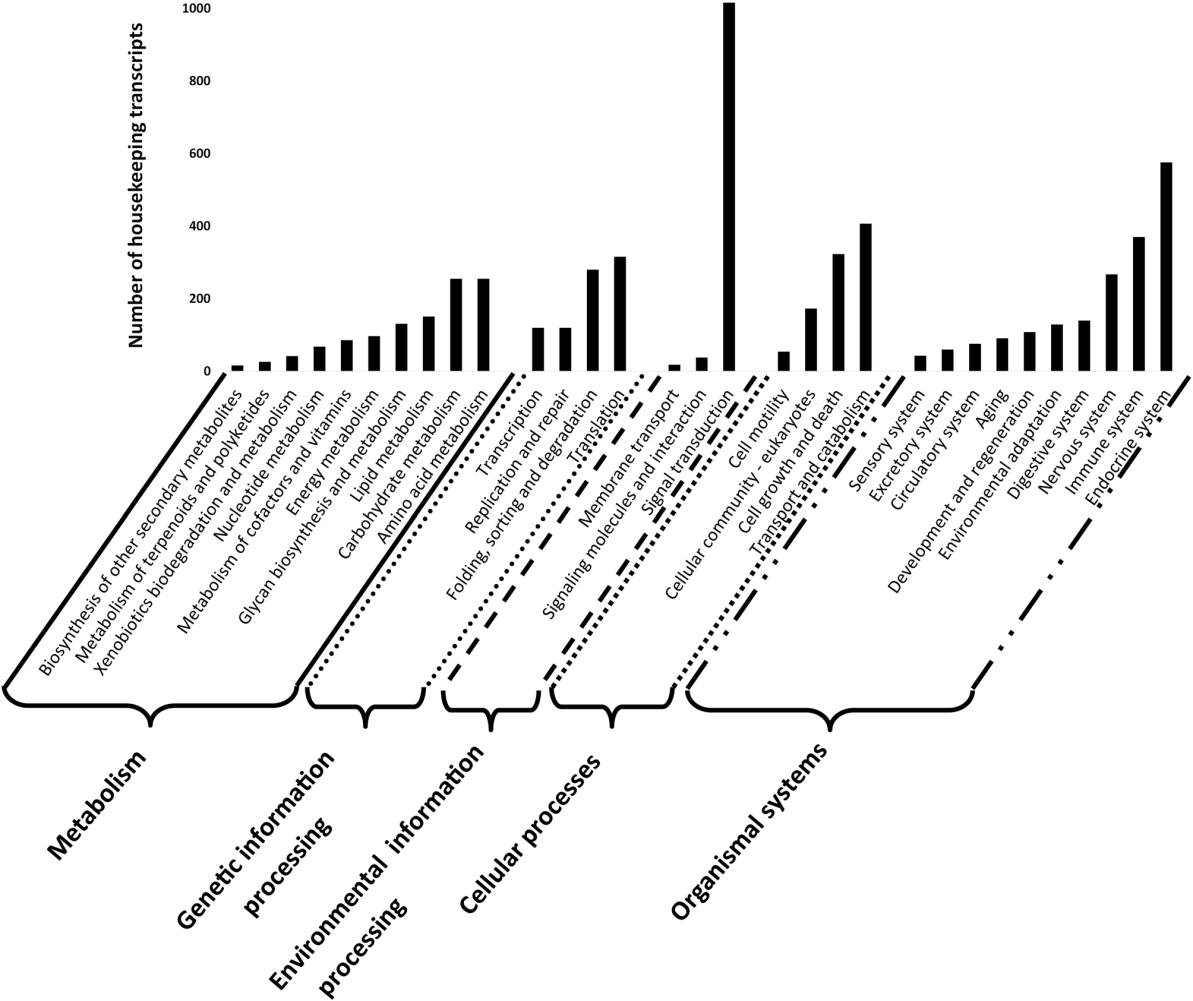
Housekeeping proteins from *Rhipicephalus evertsi evertsi* that mapped to the five groups of known housekeeping pathways defined by Kegg orthology.

The 12,206 transcripts assigned to the housekeeping class were divided into five main groups based on KEGG GhostKOALA database searches (Fig. 3). These groups relate to metabolism (n = 2120), environmental information processing (n = 1072), genetic information processing (n = 836), cellular processes (n = 961) and organismal systems (n = 1860). To infer functionality within the 5 groups, 9327 (76.4%) of the transcripts mapped to KEGG reference pathways.

Key metabolic pathways included carbohydrate metabolism (12%), amino acid metabolism (9%), and lipid metabolism (7%). While information regarding tick metabolic pathways remains limited, carbohydrate, lipid and amino acid metabolism may play a role during pathogen infection^22^.

The environmental information processing group (n = 1072) included important housekeeping processes such as signal transduction (94%), membrane transport (2%), signalling molecules and interaction (4%). Important signal transduction pathways identified were the highly conserved phosphoinositide-3-kinase-protein kinase B activated/Akt pathway (PI3K-Akt signalling pathway), the mammalian target of rapamycin (mTOR) signalling pathway, which both control cell metabolism, growth, proliferation and survival^19^ and the mitogen-activated protein kinase (MAPK) signalling pathway. These pathways are ubiquitously expressed and evolutionary conserved in eukaryotes^23^.

The genetic information processing group contained the genetic informative processes encapsulated in the Central Dogma represented by 15% transcription, 38% translation, 14% DNA replication and repair and 33% folding, sorting and degradation of proteins.

#### Secretory functional class

**Figure 4:**
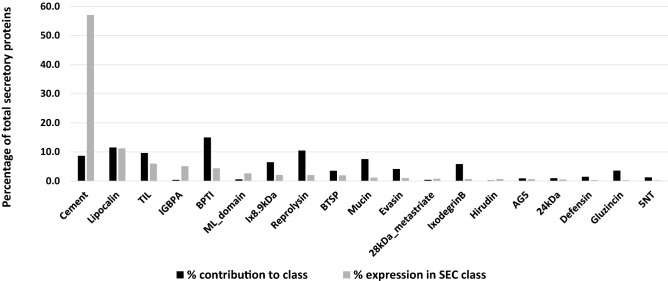
Expression analysis of the secretory (SEC) proteins in the *Rhipicephalus evertsi evertsi* transcriptome (n = 2040). The glycine-rich protein family (cement) accounted for 57% of the total expression within the class while contributing less than 10 percent of the transcripts in the class. Expression was measured by transcript per million (TPM).

Protein families within the secretory class (n = 2040) were expressed at varying levels ranging from TPM 3.59–90813 or 0.002% to 57% of the total TPM with no correlation between the number of proteins in the family and what they contribute toward total transcript expression within the class (Table 1). The largest contributor to the expression of the secreted proteins were the glycine-rich protein family (cement) representing 57% to the total transcript expression in the secretory protein class from only 177 transcripts, followed by the lipocalins contributing 11% from 235 transcripts. The remaining nine protein families had TPM values from 9525 to 1907 (6 to 1%) and consisted of a range of transcripts per family (8 to 306) (Fig. 4). These included TIL domain proteins, immunoglobulin-binding proteins (IGBPB), bovine pancreatic trypsin inhibitor (BPTI), ML domain lipid-recognition proteins, proteins belonging to the 8.9-kDa polypeptide family unique to hard ticks, reprolysin/metalloproteases, basic tail secretory proteins (BTSP), mucins and evasins. Those with the lower TPM values included proteins belonging to the 28-kDa metastriate specific protein family, Ixodegrin B, a protease inhibitor similar to the Hirudin-like superfamily, antigen 5 cysteine rich proteins (AG5), 24 kDa proteins, defensins, gluzincin, 5′ nucleotidases like apyrase (5NT) and secretory proteins with unknown functions (Table 1) (Fig. 4).

**Table 1:** Characteristics of major tick secretory protein family expression in the *Rhipicephalus evertsi evertsi* transcriptome.

Secreted protein family	Average TPM of protein family	Number of family members	Proportion of the total number of secretory proteins represented by this family (%)	Proportion of the secretory protein class expression represented by this family (%)	ID of the top expressing member in the family	TPM value of the top expressing member in the family	Proportion of the protein family represented by the top expressing member (%)
24 kDa	41.82	20	0.98	0.53	Reve_36790	138.42	16.55
28kDa_metastriate	155.61	8	0.39	0.78	Reve_9246	683.13	54.88
5NT	12.66	26	1.27	0.21	Reve_53772	126.37	38.40
7DB	4.60	5	0.25	0.01	Reve_5751	7.11	30.94
AG5	53.36	19	0.93	0.64	Reve_406	299.12	29.50
BPTI	22.74	306	15.00	4.37	Reve_16283	490.75	7.05
BPTI_lipocalin	3.59	1	0.05	0.00	Reve_50393	3.59	100.00
BTSP	43.25	72	3.53	1.96	Reve_34913	354	11.37
Cement	513.07	177	8.68	57.09	Reve_54513	13,233.53	14.57
Cys-richD2	4.74	1	0.05	0.00	Reve_14221	4.74	100.00
Cystatin	14.91	8	0.39	0.07	Reve_29872	78.52	65.83
Defensin	17.73	30	1.47	0.33	Reve_63270	89.29	16.79
EF-hand/Longistatin	40.81	1	0.05	0.03	Reve_37845	40.81	100.00
ETX_MTX2	16.75	15	0.74	0.16	Reve_40855	83.26	33.14
Evasin	18.76	85	4.17	1.00	Reve_41657	448.52	28.12
FreD	23.01	10	0.49	0.14	Reve_11395	45.42	19.74
Gluzincin	6.14	73	3.58	0.28	Reve_4761	48.74	10.87
Hirudin	270.86	4	0.20	0.68	Reve_50959	418.08	38.59
IGBPA	1014.99	8	0.39	5.10	Reve_38932	3153.12	38.83
Ix26kDa	5.15	3	0.15	0.01	Reve_46771	6.04	39.09
Ix8.9 kDa	25.26	132	6.47	2.10	Reve_16140	427.27	12.81
IxodegrinB	9.35	119	5.83	0.70	Reve_13333	147.83	13.29
Kazal	18.60	5	0.25	0.06	Reve_26944	70.44	75.75
Kazal/SPARC	18.16	2	0.10	0.02	Reve_53837	21.02	57.89
Kazal/vWf	13.30	4	0.20	0.03	Reve_58653	40.02	75.24
Lipocalin	76.20	235	11.52	11.26	Reve_17348	3439.33	19.21
Longistatin	30.90	1	0.05	0.02	Reve_56956	30.9	100.00
Metalloprotease	5.92	15	0.74	0.06	Reve_32715	34.15	38.48
ML_domain	349.40	12	0.59	2.64	Reve_19418	4106.88	97.95
Mucin	12.38	154	7.55	1.20	Reve_26068	441.03	23.13
Reprolysin	15.39	214	10.49	2.07	Reve_62060	243.17	7.38
SALP15	3.55	2	0.10	0.00	Reve_10966	3.87	54.51
Serpin	2.94	32	1.57	0.06	Reve_60207	11.99	12.76
Sphingomyelinase	3.48	14	0.69	0.03	Reve_9137	12.18	25.01
Unknown sec protein	43.08	7	0.34	0.19	Reve_42700	205.79	68.24
TCI	15.17	15	0.74	0.14	Reve_19551	105.84	46.52
Thyropin	4.98	8	0.39	0.03	Reve_37339	10.66	26.78
TIL	48.35	197	9.66	5.99	Reve_24953	1551.15	16.28
Grand total	77.98	2040	100.00	100.00

### Orthologs in the *Rhipicephalus evertsi evertsi* transcriptome for proteins with known functions involved at the tick-host interface

To date functions for ~ 120 tick proteins involved at the tick-host interface have been experimentally validated^24^. Finding such orthologs within a transcriptome is an important quality measure, since it allows assignment of function based on homology, experimentally verified functions and provide confirmation that such functional proteins are present in the transcriptomes of evolutionary related organisms. A BLASTP^14^ analysis of protein sequences with known functions followed by phylogenetic analysis allowed identification of 22 potential orthologs (Table 2).

**Table 2:** Orthologs identified in the *Rhipicephalus evertsi evertsi* transcriptome for proteins with known functions.

Protein	Function	Protein family	Orthologs in transcriptome	References
Apyrase*	ATP/ADP hydrolysis	5′-nucleotidase	Reve10069, Reve7259, Reve53772, Reve29549, Reve67417	^111^
Aas19	Platelet aggregation; Thrombin	Serpin	Reve9044	^60,112^
AaS27	Inflammation; fXIa	Serpin	Reve68878	^113^
IRS-2	Cathepsin-G	Serpin	Reve68878	^114^
Amblin	Thrombin	Kunitz	Reve18091, Reve25119	^54^
Boophilin	Thrombin	Kunitz	Reve26164, Reve34404, Reve25119	^56^
Rhipilin 1	Anticoagulant	Kunitz	Reve1377, Reve53258,	^115^
Rhipilin 2	Elastase	Kunitz	Reve35686	^116^
Longistatin*	Plasminogen activator	EF-hand	Reve56956, Reve37845	^64^
Enolase	Plasminogen receptor		Reve2489	^117^
IsMP	Fibin(ogen)olytic	Metalloprotease	Reve2893	^46^
Iris	Hemostasis	Serpin	Reve33028	^118^
HBP1*	Histamine	Lipocalin	Reve4256, Reve4504, Reve17348, Reve9406	^51^
EvasinB	Chemokines	Knottin	Reve23059	^119^
Histamine release factor	Vascular permeability	Translationally controlled tumor protein (TCTP)	Reve6137	^120,121^
IGBPA	IgG binding		Reve12277	^37^
IGBPC	IgG binding		Reve43878, Reve19418	^37^
Angiotensin converting enzyme*	Bradykinin		Reve50087, Reve2424, Reve60442	^49^
CirPT1	Complement C5	Von Willebrandt factor type C	Reve_16140, Reve18664, Reve62569, Reve34768	^52^
RmS-15	Thrombin	Serpin	Reve9044	^61^
EVA-P991	Chemokines	Evasin A	Reve41657	^122^

Those proteins with asterisks are considered orthologous to ancestral tick lineages of platelet aggregation inhibitors, blood clotting inhibitors and immune-modulators^26^. Orthologs were confirmed by phylogenetic analysis and were included if the homologs grouped within a well-defined orthologous clade with good bootstrap support that included the sequence with experimental confirmed function.

### Gender dependent transcript expression patterns

**Figure 5:**
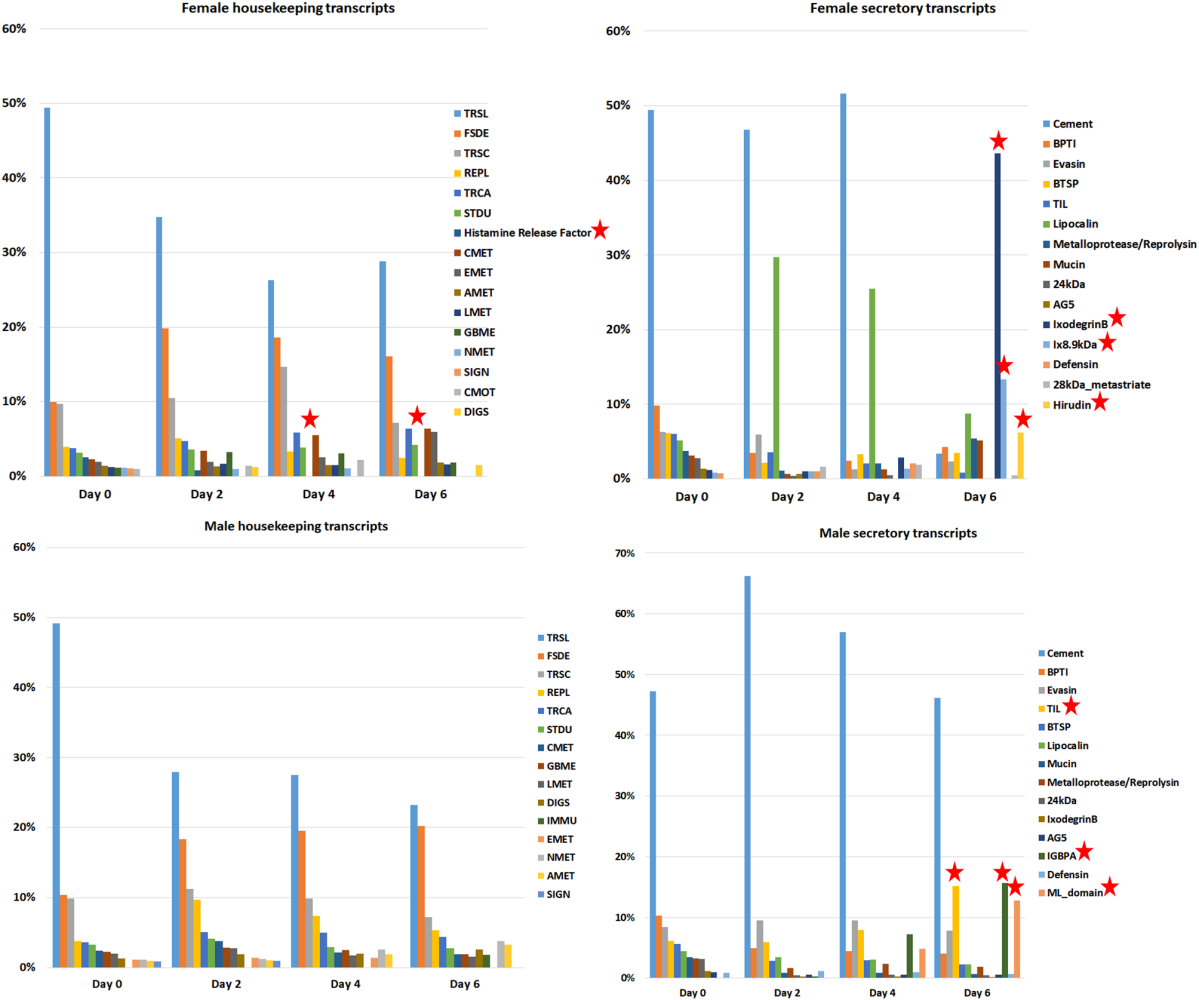
Female and male housekeeping and secretory protein expression patterns per day expressed as percentage per day based on TPM ≥ 0.5 cut off for females and males. Protein families with a significant increase or decrease in expression are indicated with stars. Represented housekeeping proteins were: translation (TRSL), folding sorting and degradation (FSDE), transcription (TRSC), replication and repair (REPL), transport and catabolism (TRCA), signal transduction (STDU), carbohydrate metabolism (CMET), energy metabolism (EMET), amino acid metabolism (AMET), lipid metabolism (LMET), glycan biosynthesis and metabolism (GBME), nucleotide metabolism (NMET), signaling molecules and interaction (SIGN), cell motility (CMOT), digestive system (DIGS).

To determine expression patterns over time for each sex, single reads for each time point were mapped so the MDS to estimate transcript expression per sampling point of unfed, early feeding, mid-feeding and late feeding (Fig. 5). The male and female transcriptomes shared 83.3% ORFs with 5.1% unique ORFs in the female transcriptome and 6% unique male ORFs. The class with unknown functions shared 4.8% ORFs between the sexes with 0.2% unique ORFs in the males and 0.6% ORFs in females.

The most abundant housekeeping classes, namely translation (TRSL), folding, sorting, degradation (FSDE) and transcription (TRSC) in both male and female transcriptomes accounted for the majority of the overall expression in each (51% female, 47% male) (Fig. 5). The housekeeping proteins involved in translation, folding sorting, degradation and transcription followed the trend of the whole transcriptome at being the most abundant and highly expressed in both the male and female salivary gland transcriptomes although there was a 5% difference in expression in the male (TRSL TPM = 98,784) and the female’s (TRSL TPM = 127,389).

The male dominant secretory protein, immunoglobulin-binding protein (IGBP), was highly expressed from only a few transcripts and accounted for 7% of the male secretory protein transcript expression. This was followed by the glycine-rich superfamily (cement), which was expressed 4% higher in the male transcriptome than in the female. The metalloproteases made up 2% of each gender’s transcriptome while the Kunitz-type inhibitors (BPTI), lipocalins and basic tail secretory protein (BTSP) families each contributed to 1% in both male and female expressed proteins with major expressed lipocalins having predicted functional folds of serotonin and histamine binding activity using the Phyre^2^ protein fold recognition server^25^. However, only 25% (18 of the 72 identified female lipocalins) had the canonical biogenic amine-binding motif that would support binding of histamine and serotonin^26^.

The initial high expression of cement by the females decreased between day 4 to 6 (~ 50% to less than 10%). The secretion of lipocalins, evasins, Kunitz-type inhibitors, TIL and BTSPs increased after day 0 and varied during the feeding period of day 2 to 4 (~ 10 to 30%) but lipocalins had a gradual decrease of expression as feeding progressed. At the day 4 to 6 feeding ixodegrin B, Ix8.9 kDa and a protein assigned to the hirudin class of anticoagulants, increased in expression value to be the most expressed proteins just before the females detached (< 10% and > 10%). In contrast, the expression of cement by the males remained relatively constant throughout the feeding period as did evasins, BPTIs and TIL with an increase of IGBPA toward day 6. Lipocalins were not as highly expressed in the males as in the females.

**Figure 6:**
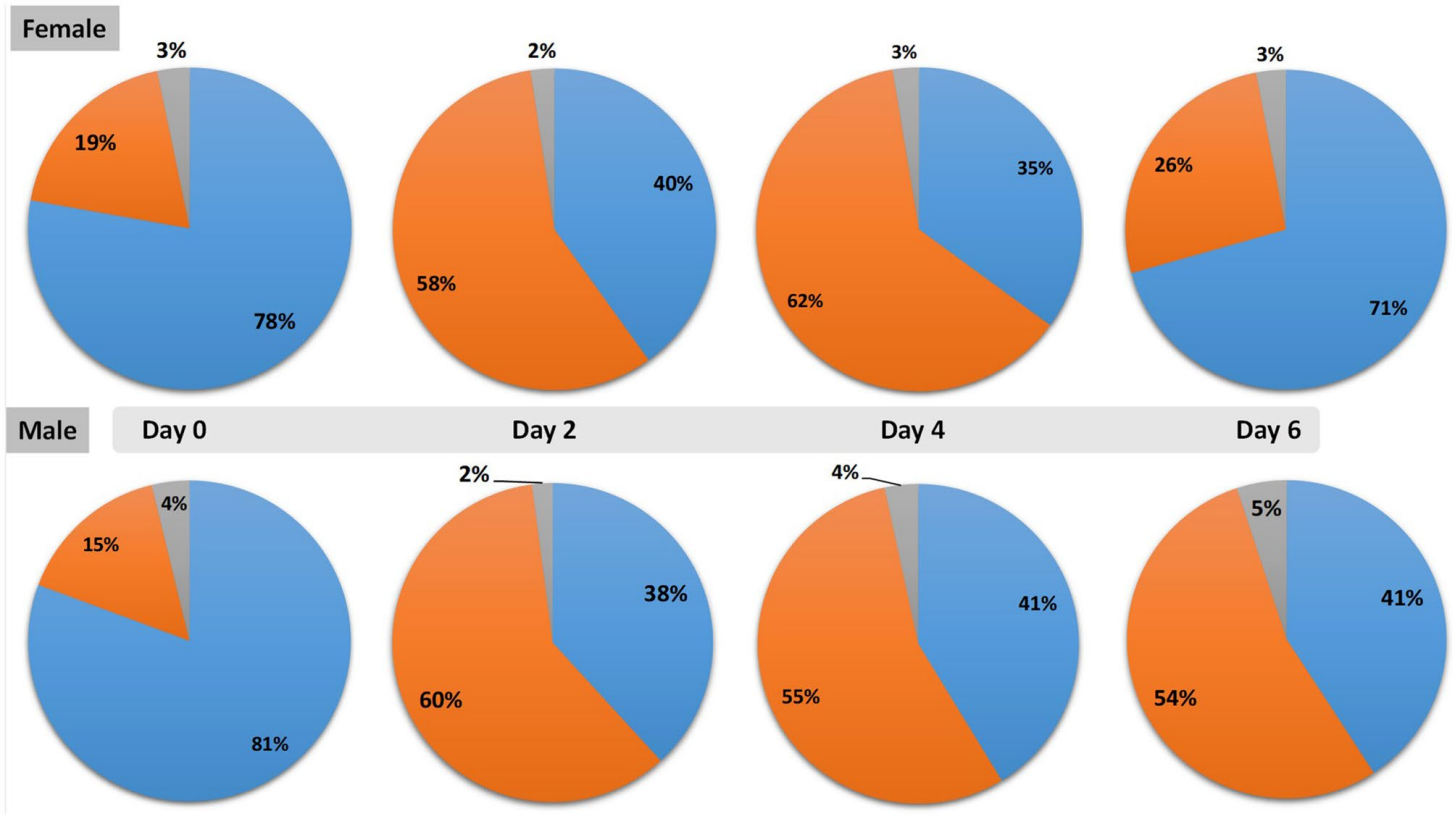
Illustrative effect of expression of the housekeeping (blue), secretory (orange) and unknown (grey) functional classes over time. Expression was measured as TPM ≥ 0.5 for both the male and female transcriptomes.

In both the male and the female transcriptome, the highest expressed housekeeping proteins (TRSL, FSDE, TRSC and TRCA) stayed constant with a gradual decrease in expression of TRSL and increase from day 2 for FSDE, TRSC and TRCA. To confirm the observation of an increase in expression during the feeding period and a down tapering toward the end, a simple illustration of the housekeeping, secretory and unknown classes followed suit (Fig. 6). Overall, certain protein families were predominantly expressed in either one of the sexes, especially in the case of the secretory proteins; the lipocalins, 28 kDa metastriate and ixodegrins appear to be more expressed in the female transcriptome.

### Differential expression between male and female transcriptomes

**Figure 7:**
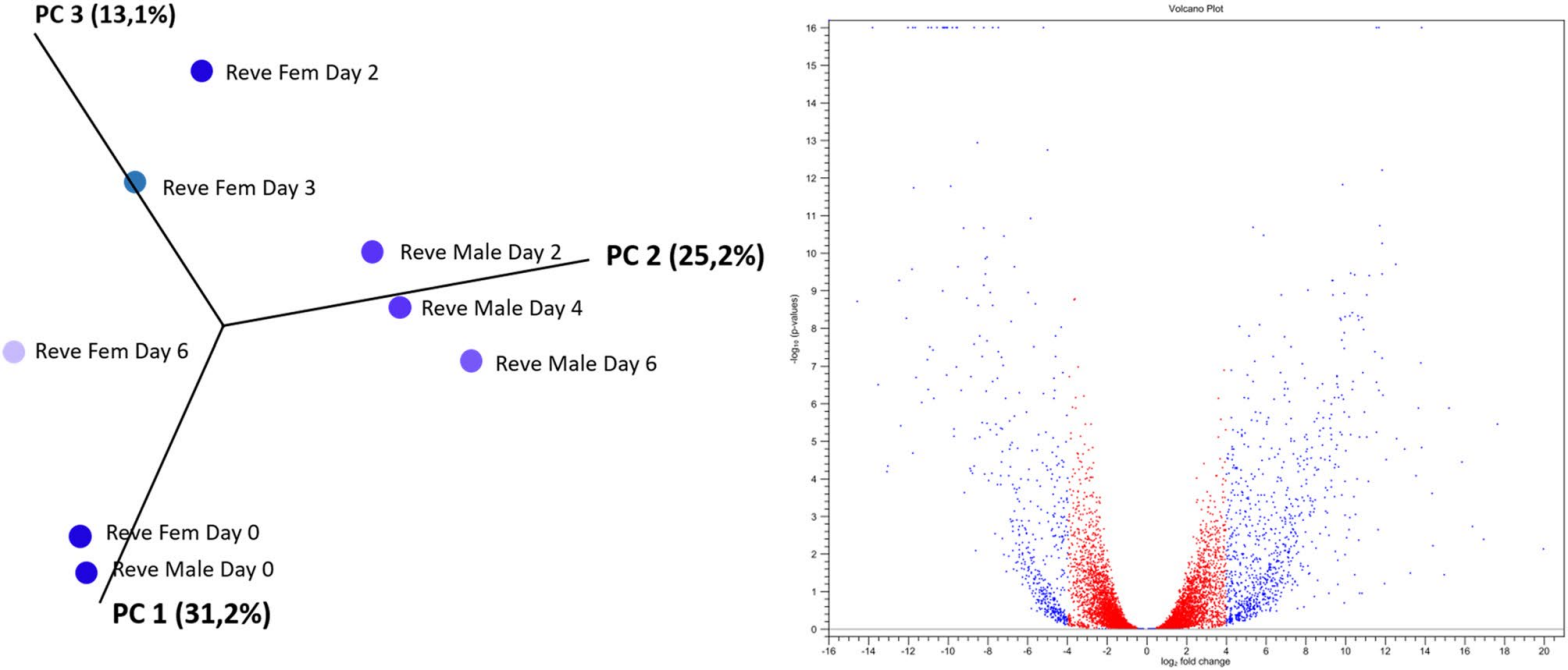
Principle component and subsequent expression analysis (volcano plot) of the male and female transcripts controlled for by day to identify differentially expressed genes over time in the male and female transcriptome. The scatter plot displays the largest variance between unfed male and female ticks (Reve Fem Day 0 and Reve Male Day 0) (31.29%) followed by the second largest variance between feeding time points of males (25.2%). The volcano plot represents the differentially expressed female and male transcripts over the feeding period as a fold change against absolute confidence. Differentially expressed transcripts were double filtered to have FDR value < 0.05 and a log2 fold change larger than 4 and smaller than − 4 (dots in blue). Those that are highly up- or downregulated are further to the right and left sides, while highly significant changes appear higher on the plot.

Principle component analysis (PCA) confirmed differential expression between male and female tick salivary gland transcripts and clustered feeding points based on similarities in their expression patterns (Fig. 7). There was a clear grouping of the unfed (day 0) males and females before changes in expression started. Males of day 2, 4 and 6 clustered together but separate from day 0, suggesting that differential expression occurs in males once feeding starts, but then stabilized. In contrast, expression patterns for the females of day 2 and 4 clustered together before a remarkable change in expression of the day 6 female salivary glands. The female differentially expressed proteins’ ranged from TPM 31,945 for a secretory Ixodegrin B-like protein to TPM 0.1 for a translational housekeeping protein. In the case of the males the highest differentially expressed protein was a ML-domain housekeeping protein (TPM 19,725) as compared to the lowest of the housekeeping proteins involved in the metabolism of terpenoids and polyketides (TPM 0.05).

The projection by the PCA analysis presented the transformation from a metabolically inactive to metabolically active tick salivary gland, observing the most variation between day 0 males and females and the rest of the male feeding stages (31.2%) with the second largest variation being between the sequential days of male transcriptome (25.2%) and the female’s (13.1%) which had a dynamic range of variance in time points sampled (Fig. 7).

Both sexes had similar patterns in terms of overall expression of protein classes however, by applying the filters to the volcano plot data (FDR < 0.05, log2 fold, − 4 to 4 expression) 3258 transcripts from the female and 2086 from the male differentially expressed transcripts were identified. Interestingly, the largest fold changes in terms of the magnitude of the difference of expression values in the group were as predicted by the principle component analysis, between proteins of the unfed and day six transcriptomes especially the housekeeping proteins (Table 3). This was even more evident in the female transcriptome. Most of these major fold changes occur from day two up to day 6 of feeding for the females with 12% for the housekeeping proteins and 20% for the secretory proteins. Similar to the females, the male’s largest fold changes of differentially expressed proteins were also between the unfed and day 6 males but had a lot less expression changes (< 10%) during feeding i.e. day 2 versus 4, day 2 versus 6 and day 4 versus 6 for both the housekeeping and secretory proteins (Table 3). This may be expected from the male transcriptome.

**Table 3:** Counts of transcripts of each class; housekeeping (HKP), secretory protein (SP) and unknown function proteins (UNK), displaying their largest fold change or mean expression value relative to time points of sampling the female and male salivary gland transcriptome based on differential expression by RNA-Seq analysis using global trimmed mean of M values (TMM) normalization.

Largest fold change between days	Housekeeping				Secretory proteins				Unknown proteins				Total number of transcripts
	Number of transcripts	Fold change	*p* value	FDR *p* value	Number of transcripts	Fold change	*p* value	FDR *p* value	Number of transcripts	Fold change	*p* value	FDR *p* value	
**Male transcript largest fold changes**													
0 versus 2	286 (14%)	− 78.806	0.0016	0.0028	289 (14%)	− 775.167	0.0003	0.0005	23 (1%)	− 129.852	0.0018	0.0030	598
0 versus 4	304 (15%)	− 19.215	0.0021	0.0037	125 (6%)	− 482.646	0.0010	0.0017	27 (1%)	− 38.562	0.0034	0.0057	456
0 versus 6	389 (19%)	− 979.352	0.0014	0.0024	183 (9%)	− 4409.253	0.0005	0.0009	43 (2%)	− 1691.050	0.0005	0.0009	615
2 versus 4	110 (5%)	− 6.487	0.0030	0.0051	19 (1%)	48.490	0.0028	0.0048	5 (0%)	17.587	0.0003	0.0006	134
2 versus 6	140 (7%)	− 18.254	0.0027	0.0047	14 (1%)	60.135	0.0047	0.0074	5 (0%)	− 25.472	0.0068	0.0116	159
4 versus 6	103 (5%)	− 16.575	0.0042	0.0072	8 (0%)	39.759	0.0018	0.0031	13 (1%)	35.633	0.0055	0.0094	124
Total number of transcripts	1332				638				116				2086
**Female transcript largest fold changes**													
0 versus 2	211 (6%)	− 63.870	0.0009	0.0012	71 (2%)	− 6557.445	0.0001	0.0001	18 (1%)	− 1295.401	0.0001	0.0002	300
0 versus 4	264 (8%)	− 95.169	0.0009	0.0013	104 (3%)	− 3077.285	0.0001	0.0002	15 (0%)	− 74.198	0.0012	0.0017	383
0 versus 6	497 (15%)	− 147.703	0.0005	0.0006	344 (11%)	− 292.683	0.0004	0.0005	46 (1%)	46.014	0.0002	0.0003	887
2 versus 4	119 (4%)	39.673	0.0029	0.0040	17 (1%)	− 882.441	0.0025	0.0033	8 (0%)	10.090	0.0037	0.0051	144
2 versus 6	395 (12%)	587.418	0.0009	0.0011	636 (20%)	2193.794	0.0002	0.0003	43 (1%)	2104.814	0.0000	0.0000	1074
4 versus 6	198 (6%)	145.625	0.0023	0.0031	255 (8%)	1724.236	0.0002	0.0002	17 (1%)	74.372	0.0046	0.0061	470
Total number of transcripts	1684				1427				147				3258

The percentage contribution to female and male differentially expressed transcripts are indicated in parentheses. The fold-change, *p* value and false-discovery rate *p* value (FDR *p* value) are also indicated as averaged values for the time point.

By removing the shared transcripts between the male and female differentially expressed transcripts, the males had 968 differentially expressed transcripts with 77% housekeeping, 16% secretory and 7% ‘unknown’ hits while the females had 2140 of which 4% were ‘unknown’, 44% secretory and 52% housekeeping.

**Figure 8:**
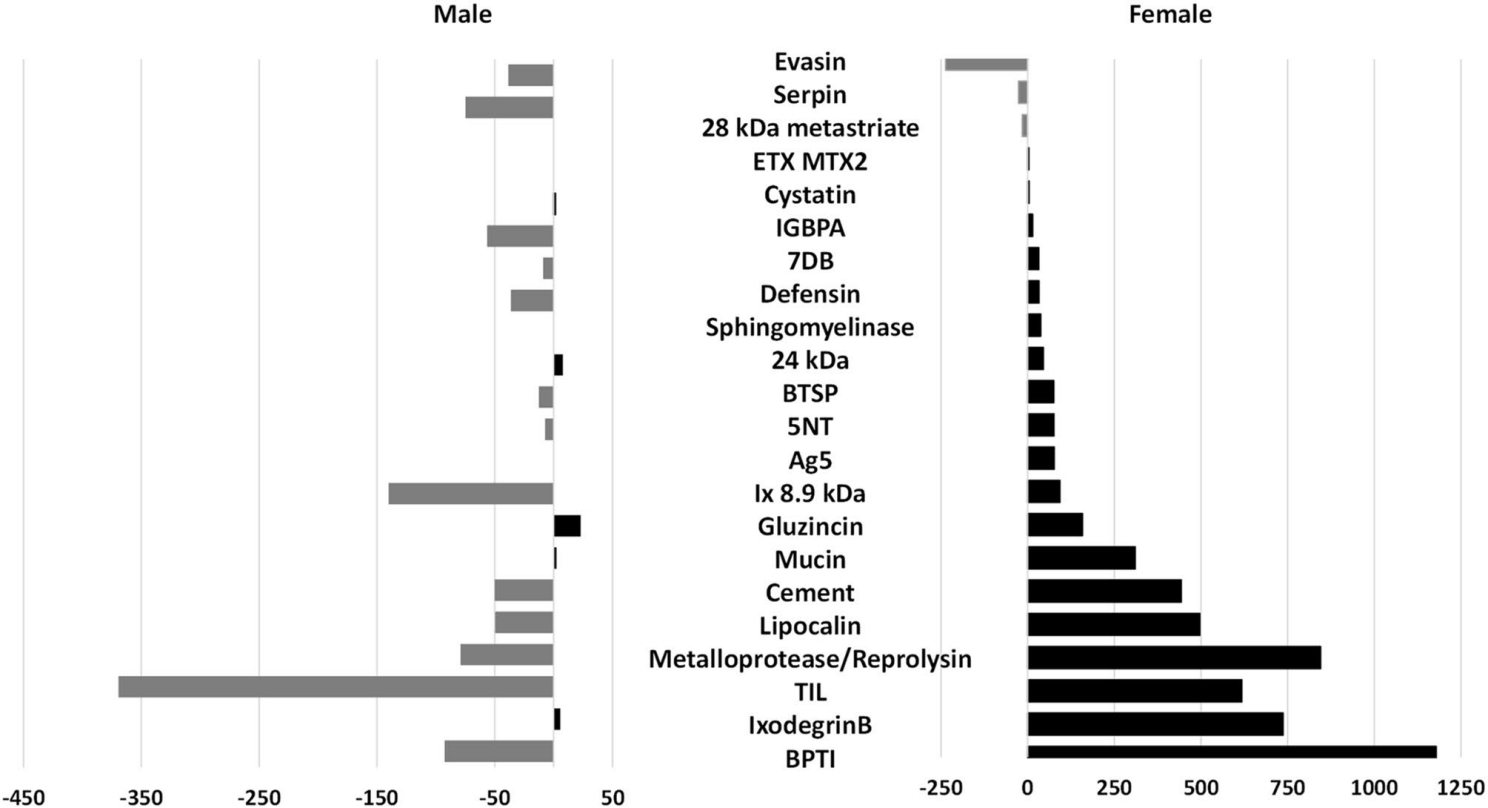
Differentially expressed secretory proteins in the male and female transcriptome with the highest significance as related to fold change over time compared to protein abundance as counts. Positive log2 fold changes are indicative of upregulation while negative log2 fold changes indicate downregulation.

These differentially expressed proteins, in both the male and female transcriptomes, with the highest significance in relation to fold change over time, measured as log2 fold change, were of particular interest (Fig. 8). The visual trend in the differentially expressed proteins of the male transcriptome is one of downregulation as opposed to the female’s upregulation dominated differentially expressed proteins. The majority of the differentially expressed transcripts were of the secretory class in both sexes. It would also support the observation of the high fold changes from day 2 to 6 for the females. Almost all secretory proteins are differentially expressed to a lesser degree in the males. This included the previously highly expressed cement, evasin, TIL proteins, IGBP and BPTIs. Gluzincin and the 24 kDa proteins displayed some upregulation. The females on the other hand, displayed a range of upregulated differentially expressed proteins with a downregulation of their evasin protein secretion. Those differentially expressed protein families with significant upregulation in the female salivary glands were the BPTIs, Ixodegrin, TIL, lipocalins, cement and mucins.

## Discussion

The aim of the current study was the de novo assembly and annotation of a salivary gland transcriptome for *R. evertsi evertsi* representing various feeding stages that included unfed, early feeding and late feeding of males and females. Sequence data for *R. evertsi evertsi* have been oddly scarce since it has the widest geographical range of *Rhipicephalus* in Africa^24,27^, with only 153 nucleotide and 28 protein sequences available in GenBank before the start of the current study (confirmed on 13 June 2020). This study improves on this shortfall by contributing 15,115 annotated proteins. The assembled transcriptome quality in terms of read mapping-based assessment and reference sequence-based assessment, even after rigorous removal of duplicated sequences to render a minimal dataset, indicated it to be representative of the sequenced reads with a comparable level of accuracy and completeness. It is comparable to available salivary gland transcriptomes from other genetically related ticks. Most of the transcripts (95%) could be annotated or at least assigned to a functional class which left 6% with an unknown status contributing ~ 3% (n = 869) of the overall expression in the salivary glands. Functional classes represented those transcripts involved in housekeeping of the cell, secretory proteins with the presence of signal peptides, or of unknown function. Those with an unknown status showed homology to other unknown proteins in the database based on their BLAST E-values. Putative functions were attributed based on previously characterized proteins in publicly available databases. Experimental confirmation of such functions needs to be performed in future.

The housekeeping functional class contributed to the majority of the composition of the transcriptome (80%) but with lower expression (51%), while the secretory protein functional class represented only 14% of the transcriptome but 46% of the total coverage. This was comparable to the overall description of the secretory category of other salivary gland transcriptomes from *Amblyomma americanum*, *Ixodes ricinus, R. appendiculatus, R. microplus, R. pulchellus*, *R. sanguineus* and *R. zambeziensis* (between 12 and 63%)^5,6,16,18–21^. Nonetheless, the transcriptomes displayed a dynamic range of expression with a few transcripts responsible for most of the expression in the salivary glands. The unknown class was not analysed further although it may contain housekeeping proteins. The housekeeping classes is close to the ~ 7000 shared core housekeeping genes previously predicted as part of the core eukaryotic set of orthologous genes^28^, suggesting that the *R. evertsi evertsi* transcriptomes represent a large portion of the expected housekeeping genes.

### Housekeeping functional class

More than 15% of the housekeeping transcripts that could map to KEGG reference pathways were responsible for environmental information processing relating to membrane transport, signalling transduction, signalling molecules and interaction. These processes rely on proteins (receptors) and their chemical signals (ligands) to regulate a cell’s internal microenvironment in response to external changes through the movement of molecules or fluids across membranes. For example, the active secretion of saliva from the salivary gland is controlled by dopamine (ligand) which activates two signal transduction pathways for salivary secretion: the cyclic adenosine monophosphate (cAMP)-dependant signal transduction pathway that leads to fluid secretion and the calcium-dependant signalling pathway that activates prostaglandin E2 production which ultimately also leads to protein secretion in tick saliva^29^. Orthologs for the dopamine D1-like receptors were present in this transcriptome^30^. Furthermore, osmoregulation and salivation via the salivary glands is a continuous process controlled by a complex neural and endocrine system^31^ that requires concentrating the nutrients from the blood meal and pumping approximately 75% of ingested water and ions back into the host. The major intrinsic protein (MIP) neuropeptide and respective G-protein coupled receptors forms part of osmoregulation^32^. MIP and its predicted G-protein coupled receptor were also identified in the current transcriptome. MIP and its receptors function in the modulation of membrane channels selectively transporting water and ions in and out, as well as between cells assisted by aquaporin. More recently, aquaporin’s involvement was extended to inflammatory processes in humans as reviewed^33^. This function could be extended to the complex mechanisms regulating inflammation in the tick-host feeding site and possibly the complexity of molecules needed in tick saliva to regulate host aquaporin signalling pathways during inflammation. Additional research into aquaporin’s regulatory mechanisms might be an alternative therapeutic target in the prevention or treatment of inflammation.

The majority of transcripts involved in genetic information processing were associated with mRNA translation to proteins (38%). This is the protein synthesis function of the ribosomes in the cytoplasm or endoplasmic reticulum after gene coding DNA has been transcribed to mRNA in the cell nucleus. It is a continuous process evident by the differential expression during feeding and illustrative of how the tick is able to constantly adapt to a changing feeding site and host immune defences. These housekeeping proteins (41%) contribute to the successful continuous feeding by the tick and are supported by key metabolic pathways with the majority of the transcripts involved in amino acid metabolism, carbohydrate metabolism and lipid metabolism.

### Secretory functional class

The secretory functional class represented 46% of the overall expression in the transcriptome with the most abundant secretory protein transcripts being the BPTI (15% of the transcripts of the secretory protein class contributing ~ 4% to the secretory class and 2% of overall expression), the lipocalins (11.5% of the transcripts of the secretory protein class contributing ~ 11% to the secretory class and 5% of overall expression) and metalloprotease/Reprolysin families (10.5% of the transcripts of the secretory protein class contributing ~ 2% to the secretory class and 1% of overall expression). This was to be expected since these are major protein families that expanded due to gene duplication in the evolution of blood-feeding tick lineages^2,3^. To put the secretory protein families into functional context a brief description of each is provided with regard to known functions.

#### Cement formation

The dominantly expressed glycine-rich protein family accounted for 26% of the total expression and 57% in the secretory class alone. Glycine-rich proteins are involved in formation of the cement cone^34^. The dominant expression profiles of glycine-rich proteins have been noted before^16,21,35,36^.

#### Immunoglobulin scavengers

The male dominant IGBPA transcripts accounted for 2% of the total expression and 5% of the secretory protein class alone. Immunoglobulin-binding proteins have orthologs in all ixodid ticks, but vary in size between species and time of expression during feeding^37,38^. IGBPAs are essential proteins with the purpose of the inactivation of host IgG, otherwise detrimental to tick survival, while aiding its secretion back into the host^39^. This is probably an adaption to their extended periods of feeding and being exposed to the onslaught of the host’s immune system as compared to the shorter feeding strategies of soft ticks. Orthologs have been found for at least IGBPA and IGBPC in this *R. evertsi evertsi* transcriptome.

#### Cytokine storms and the evasins

Inflammation is driven by host chemokines, referred to as ‘the cytokine storm’^40^ and play an important role in recruiting immune cells to the feeding site, resulting in inflammation and possible tick rejection^41^. Evasins inhibit chemokine function and a large array of inhibitors has been found in ticks^42^. In *R. evertsi evertsi* a large number of the evasin A and evasin B class were found in the transcriptome. Orthologs for EVA-P991 and evasin B were detected.

#### Antimicrobial activities and TIL domain containing proteins

The TIL domain containing proteins (peptidase inhibitors) were described from mosquitoes, hard and soft ticks^43^ including the transcriptomes of the non-hematophagous *Antricola delacruzi*^44^. These transcripts displayed a steady decrease in the female transcriptome. However, TIL protein expression increased in the male transcriptome toward the end of feeding after a relatively constant expression thereof during the preceding days. The function of these proteins suggest a role in tick innate immunity and an antimicrobial function in preventing the tick from being infected after feeding^45^.

#### Fibrinogenolytic and immunomodulatory metalloproteases

Even though the functionality of many of the metalloproteases are unknown, those isolated and characterized from *Ixodes scapularis* targeted fibrin and fibrinogen, thereby preventing blood clotting or dissolving formed blood clots^46^, affect inflammation and wound healing by degrading integrin^47^. An ortholog to the fibrinogenolytic metalloprotease was found in the *R. evertsi evertsi* transcriptome. Other authors reported this protein family to target the innate immune response, reduce pain and cause vasodilation through the degradation of bradykinin^48^. An ortholog for angiotensin-converting enzyme has been found that may target bradykinin^49,50^.

#### Anti-inflammatory lipocalins

Proteins involved in the regulation of inflammation include proteins that minimize itching by targeting biogenic amines such as the histamine-binding lipocalins^48,51^. The lipocalin family of proteins have diverse functions from modulating host immune responses by scavenging complement C5, leukotriene-C4, -B4, histamine and serotonin, to the inhibition of blood-clotting through the prevention of platelet aggregation. Orthologs has been found for histamine-binding protein 1 specific to females from *R. appendiculatus*. These orthologs were also specific to females in the current study. However, given the large number of lipocalins found in the salivary glands other functions may be expected.

#### Complement inhibitors

Recently, complement inhibitors have been found for *R. appendiculatus* that specifically targets complement C5 to control complement-mediated inflammation^52,53^. Complement inhibitor (CirPT1) presents the knottin fold (inhibitor cysteine knot (aka ICK or Knottin)), while *R. appendiculatus* complement inhibitor (RaCI) presents a unique fold that resembles the disulphide-rich structure of small toxins from snake venom^53^. Orthologs for CirTP1 were present but not RaCI.

#### Blood clotting inhibitors

The BPTI family are slow tight binding Kunitz proteins preventing blood clotting by inhibiting thrombin. Known thrombin inhibitors are Amblin, Variegin, Boophilin, Americanin, Savignin and Ornithodorin^54–59^. Orthologs for Amblin, Boophilin and Rhipilin-1 were present in the *R. evertsi evertsi* salivary transcriptome. Other proteins that may also target thrombin include serpins such as Aas19 and RmS-15^60–62^, for which orthologs were found in the transcriptome. Longistatin act as plasminogen activator and enolase as plasminogen receptor, leading to formation of plasmin (fibrinolytic enzyme) that result in feedback inhibition of thrombin via a feedback loop of the extrinsic or tissue factor pathway^63,64^. Orthologs for both proteins were present. This would indicate that targeting of the blood-clotting cascade is an important part of the feeding strategy for this tick species.

#### The basic tail secretory proteins (BTSP)

The basic tail secretory proteins are lysine-rich at the C-terminus and were well represented in other transcriptomes^65,66^. One protein was shown to exhibit anticoagulant function for the inhibition of factor Xa^67^. The initial high expression of BTSP and subsequent decrease is similar to what was found for *I. ricinus*^68^.

The involvement of a protein similar to hirudin with potent thrombin inhibiting activity and ixodegrin B, which is only expressed in tick salivary glands in the late phase of feeding is indicative of a greater prevention of platelet aggregation which would suit the purpose during the engorgement phase of feeding from the blood pool. However, they are related to lectins which may also be indicative of a function in the tick innate immunity^68^.

#### Paralysis toxins

BLASTP^14^ analysis of the 18 available holocylotoxin genes in Genbank^69^ (the paralysis-causing toxins from *Ixodes holocyclus*), found no homologs. A PSI-BLAST analysis to identify its novel inhibitory cysteine knot (ICK) toxin fold^70^, as performed previously for holocyclotoxin^12^, retrieved all family members but did not detect any homologs. It was suggested that the ICK fold is related to the 5.3 kDa family^66^. No members of the 5.3 kDa family has been detected in the *R. evertsi evertsi* transcriptome. However, the small size and high genetic divergence of the holocyclotoxins may prevent identification of toxin orthologs at present. Even so, the availability of a salivary gland transcriptome may in future enable identification of a purified toxin using proteomic methods.

### Differential expression

The differentially expressed secretory proteins, especially the multi-gene families, increased to 16% expression (compared to the initial overall 10%) and the housekeeping differentially expressed proteins decreased to 80% compared to the initial 87% overall expression. Most female differentially expressed transcripts were upregulated as opposed to those from the males, which were downregulated if assuming that they were upregulated during the initial stages of feeding. This was similar to what has been reported for *R. pulchellus* and *R. zambeziensis*^19,21^, but contradictory to *R. appendiculatus*^16^. Feeding processes between day 2 and 6 (day 3 to 4) seem to be characterized by an increase in differentially expressed transcripts as compared to the earlier and later feeding stages and was similar to expression patterns for nymphs of *I. scapularis*^71^. This period coincides with the start of the rapid engorgement phase which overlaps with the proliferative and remodelling phase of wound healing from day 3 after attachment^1^. The process of angiogenesis (the formation of new blood vessels and granulation tissue) also occurs during the proliferative phase of wound healing. The saliva of ticks therefore has potent cell proliferation and angiogenesis inhibitors, such as calreticulin^72^ and troponin-I^73,74^ to prevent wound healing and contraction. Calreticulin transcripts were present within the *R. evertsi evertsi* transcriptome as well as transcripts with the novel tropomodulin domain. Tropomodulin is a tropomyosin regulatory protein also identified in *R. sanguineus, Amblyomma triste*, *Amblyomma sculptum, Amblyomma maculatum, Hyalomma excavatum, I. scapularis and R. pulchellus*^19,35,75–79^.

The differential expression observed during progression of feeding time resonate with the concept of “sialome switching” where ticks changes their salivary gland protein expression patterns to evade the hosts immune system or as response to specific host species and environmental cues^5,7,80^. As more differential expression studies are performed this is becoming more apparent^18,20,81^. Differential expression as means to increase local concentrations at the feeding site and in response to the changing feeding site during the prolonged feeding of ixodids has also been considered^24^.

### Male and female expression patterns

Because tick attachment and feeding is not a synchronized process, graphical illustration and analysis of transcripts by means of a principle component analysis and volcano plot allowed visualization of temporal changes in transcript expression over time in both male and female transcriptomes. Gender biased expression in response to blood feeding was confirmed with differential expression analysis confirming nearly half (~ 44%) of the female differentially expressed transcripts belonging to the secretory class, similar to other reports^5,6^. For both sexes, the glycine-rich cement expression was the most dominantly expressed per day. This group of proteins is exclusive to the ixodid lineage, with the exception of the *Ixodes* genus, and is important for tick attachment during feeding^1,48,82^. The current study’s observation of the increase in spatiotemporal transcript expression for female tick salivary gland glycine-rich transcripts up to day 4 of feeding suggests a feeding induced function for maintaining the integrity of the tick-feeding site as suggested before^36^. The function of the glycine-rich transcript expression as observed in the current and other studies with regard to the male expression patterns that remain relatively constant during the feeding process^16,19,83^, remains to be tested since this presumably does not function in cement cone maintenance. The time point expression profiles of the males and females, as obtained by pooling the salivary glands of males and females of each time point together and mapping each time point’s reads to the assembled transcriptome, revealed gender dominated secretory proteins as was found in other transcriptomes^5,6,16,21,84–87^.

Focusing on transcriptional response during tick feeding and large expression differences, the male dominated IGBPA appear to drastically increase after day 2 of feeding, to a lesser degree so for trypsin inhibitor-like (TIL domain) transcripts, while evasin expression remained relatively constant. The confirmation of male dominated expression of IGBP in the salivary glands conformed to previous studies on ixodid tick expression profiles of these proteins^37,38,88^, suggesting males have a biological support role during feeding of the female ticks. With early female feeding (day 0 to 4) bovine pancreatic trypsin inhibitor (BPTI), evasin, lipocalins and BTSP dominating salivary gland expression. From day 4 to the end of feeding (day 6) which coincides with the rapid engorgement phase, ixodegrin B, Ix 8.9 kDa and a protein similar to hirudin started dominating expression along with lipocalins. They are also the families that are differentially expressed as feeding progress.

Previous studies on the proteome of the spermatophore revealed ML-domain containing proteins, TIL domain proteins and immune proteins hypothesised to be important in sperm motility and mating^89^. The observation of the increase in expression patterns of these protein families from day 4 to day 6 of feeding males might associate these proteins with the concept of male seminal fluid if mating occurred with the rapid engorgement phase of the female (from day 4 onward). These families have also been implicated in tick innate immunity, protease inhibitors, antimicrobial peptides and venom toxins^1,90^. Other studies also noted the expression of serine proteases, cystatins, immune proteins and metalloproteases as potential seminal fluid proteins^19,81,91^. However, these proteins were not annotated as ‘seminal proteins’ hindering possible orthologous comparisons between species. In the current transcriptome using a NCBI conserved protein domain search, proteins relating to male reproduction were identified. These included transcripts coding for a zonadhesin-like protein, which is a sperm-specific membrane protein associated with cell adhesion consisting of several TIL domains and an uncharacterised spermatogenesis-associated protein 20 from the conserved YyaL, SSP411 protein family, containing thoiredoxin and six-hairpin glycosidase-like domains. In humans and rats it is expressed in the testis in an age-dependent manner with SSP411 mRNA increasing during spermatogenesis^92^. A vesicle-associated membrane protein from the MSP (Major sperm protein) domain important for sperm motility also formed part of the male expressed transcriptome^93^. Notably, these proteins were present in the male transcriptome but not differentially expressed.

## Conclusions

The advances in next generation sequencing form the baseline towards functional genomic research especially in the case of a non-model organism, like *R. evertsi evertsi*, where genetic information is limited with no reference genome or proteome to annotate putative identified genes with little or no homology to other model-organisms. The current study represents the first de novo assembled transcriptome of the salivary glands from *R. evertsi evertsi* with time-dependant gender-specific transcript expression. The transcriptome was constructed using Illumina sequenced reads from unfed to representative feeding time phases of both male and female salivary glands. Unique salivary gland expression patterns in secretory protein families were observed for both sexes, the majority with unknown function, highlighting the shortfalls in tick feeding biology. This transcriptome will contribute to the increasing number of known tick transcriptomes that enabled identification of tick proteins, improving our basic understanding of tick biology and salivary gland function evident by the orthologous transcripts and those differentially expressed during male and female feeding. The identification of platelet aggregation inhibitors, blood clotting inhibitors and immune-modulators orthologous to the ancestral tick lineages confirm a common tick ancestor with similar functions than that of *R. evertsi evertsi* adding to our insight to their evolutionary salivary gland protein biology. Not having biological replicates might influence statistical variation when doing analysis across groups. However, the technology seems robust enough to be able to deduce expression trends in the *R. evertsi evertsi* transcriptome that is comparable to existing transcriptomes. Including biological replicates will offer stronger support for further statistical data analysis. It should also be kept in mind that trying to elucidate the molecular basis of tick paralysis, as possibly caused by *R. evertsi evertsi* is likely obscured by the pharmacological intricacies of tick saliva. Nevertheless, a transcriptome of this sort, sampled over time during feeding, contribute data of importance into salivary gland biology and blood feeding physiology.

## Methods

### Ethics approval

Animals used during this study were housed in the East Coast fever quarantine stable complex of the Agricultural Research Council-Onderstepoort Veterinary Research (ARC-OVR). The use of animals was approved by the ARC-OVR animal ethics committee (AEC 01.15), the animal ethics committee of the University of Pretoria (V023-16) and permission to do research in terms of Section 20 of the Animal Diseases Act, 1984 of South Africa (No: 12/11/1). All animal experiments were performed in accordance with relevant animal ethics guidelines and regulations and adhering to ARRIVE guidelines.

### Tick feedings

The ARC-OVR *R. evertsi evertsi* colony was expanded from field collected engorged females from the farm Uitspanning, Amsterdam, Mpumalanga province, South Africa in 2013. This 7-year-old colony is maintained under laboratory conditions as described^94^. Samples for the current study were obtained in 2017. For this study, female ticks were fed to repletion in the presence of males on the back of South African meat merino sheep weighing 92 kg at a ratio of 1.7 ticks/kg bodyweight with defined protocols^94^.

### Sample preparation for RNA isolation

Twenty male and females were removed, respectively, at intervals during feeding to recover material representing unfed (day 0 weighing < 5 mg), early feeding (day 2 weighing 6–15 mg for females and < 5 mg for males), mid feeding (day 4 weighing 16–23 mg for females and ~ 5 mg for males) and late feeding (day 6 weighing > 24 mg for females and ~ 7 mg for males) ticks. Salivary glands were dissected out within two hours of tick removal, cleaned from other internal tissues and placed into at least ten times its volume RNAlater (Qiagen, AMBION, Inc., Austin, Texas), and left overnight at 4 °C before storing at -70 °C. Each day’s samples were pooled by gender to render one male (40 glands) and one female (40 glands) sample per time point. Salivary glands were suspended in 600 µl RLT buffer (RNeasy Protect Mini Kit) per 20 mg tissue and total RNA extracted using the RNeasy Protect Mini Kit (QIAGEN Group). Residual genomic DNA was removed with *DNase I* digestion (QIAGEN Group). Total RNA quantification and assessment of the integrity of RNA was done using the Qubit fluorometer 2.0 (Life Technologies, Carlsbad, CA) and the Agilent Bioanalyzer 2100 (Agilent Technologies, Santa Clara, CA).

### Library construction and next generation sequencing

Libraries were prepared for sequencing using 1–1.5 µg purified total RNA and the TruSeq RNA sample Preparation kit (Illumina, San Diego, CA) with modifications. This comprised isolation of poly-A mRNA, fragmentation (for 3 min), conversion to double stranded cDNA, adaptor ligation and PCR amplification for 12 cycles. The libraries were size selected using agarose gel electrophoresis to 450–1200 bp. Bands were excised, purified and sequenced at the Biotechnology Platform of the Agricultural Research Council (South Africa) using the Illumina MiSeq system (300 bp × 300 bp). Raw sequence reads were submitted to Genbank under Bioproject PRJNA670032 and the TSA database with numbers SAMN16482386, SAMN16482387, SAMN16482388, SAMN16482389, SAMN16482390, SAMN16482391, SAMN16482392 and SAMN16482393. The final ORFs can be found under the same Bioproject number.

### Transcriptome assembly, extraction of ORFs and quality assessment

A multiple kmer, multi-algorithm, multi-dataset approach was used for the assembly. This entailed the use of three short read assemblers: Minia^95^ and Trinity^96^ along with CLC Genomics Workbench [CLC Genomics Workbench 7.0 and 12.0 (https://www.qiagenbioinformatics.com/)]. Trinity v2.4.0 was used with default parameter settings using a kmer size of 25. Minia v3.2.4 was used with kmer sizes in step sizes of 10 starting at 29 up to 99 (8 assemblies). CLC kmer sizes were used in step sizes of 5 starting at 15 up to 60 and an additional assembly using kmer 64 (11 assemblies). CLC assembly parameters were set up as follows: mismatch cost-2, insertion cost-3, deletion cost-3, length fraction-0.9, similarity-0.9, minimum contig length-240, kmer size-variable, bubble size-automatic. The number of assemblies produced in this manner totalled 66 for Trinity, 528 for Minia and 726 for CLC Genomics Workbench. ORFs were extracted using a Perl-script and chimeric and duplicate sequences removed by clustering at 90% identity using CD-HIT v4.6.4^13,97^. The All-Single dataset was mapped against the clustered ORFs using CLC Genomics Workbench and ORFs with RPKM > 5 were selected. Further reduction was performed with BLASTX analysis against the ACARI database and hits with E-values below 0.004 were selected for further analysis. To identify additional duplicates, translated ORFs with the same BLASTP^14^ hits were pairwise compared and analysed against the ACARI database using BLASTP^14^ analysis to identify ORFs with intact domains with misassembled regions. ORFs were retained that showed intact domains and highest coverage in TPM value that could be unambiguously assigned to evolutionary conserved homologs. This enabled construction of a final transcriptome database with high confidence. The transcriptome quality was measured for accuracy, completeness, contiguity and chimerism using the Benchmarking Universal Single-Copy Orthologs (BUSCO v3) approach^15^.

### Transcriptome annotation: identification of tick secretory and house-keeping proteins

Translated protein sequences were analysed against the ACARI protein database using BLASTP^14^ and hits with a cut off of E-value ≤ e−4 were retained. The ACARI (mites and ticks) protein database is an in-house curated database of protein sequences derived from Genbank or VectorBase^28,98^ and the EuKaryotic Orthologous Groups (KOG) dataset^99^. Where no protein sequences were available, nucleotide, expressed sequence tag (EST) or assembled contigs from the short read archive (SRA) were retrieved from Genbank or VectorBase and ORFs were extracted as described above and annotated as TPA (third party annotation). The ACARI database is curated based on Kyoto Encyclopedia of Genes and Genomes (KEGG)^100^ for the annotation for house-keeping pathways along with KEGG Automatic Annotation Server (KAAS) and GhostKOALA to assign KEGG orthology (KO) identifiers to the transcripts^101^. Annotations from Acari genome projects and PSI-BLAST analysis^102^ using tick secretory proteins from literature were used to identify secretory protein families. This first pass analysis assigned proteins to house-keeping or secretory classes based on sequence homology. To confirm this, ORFs were submitted to the SignalP 4.1 Server^103^, TMHMM server v.2.0^104^ and Phobius^105^. Orthologs to proteins with experimental determined functions were identified using BLASTP^14^ analysis followed by multiple alignment using MAFFT v7.311^106^ and phylogenetic analysis using IQ-TREE v1.6.12^107^. To identify reciprocal best hits, BLASTP^14^ analysis was preformed using pairwise analysis and a final cutoff value of e-6^108^. Results from the analyses were presented using InteractiVenn^109^.

### Male and female expression patterns over time

The single reads of each time point (Day 0, Day 2, Day 4 and Day 6) for male and female salivary glands were mapped against the de novo assembled transcriptome using CLC Genomics Workbench (12.0), using the RNAseq module. This was obtained using default parameters of Mismatch cost = 2, Insertion cost = 3, Deletion cost = 0, Length fraction = 0.8, similarity fraction = 0.9, both strands specific and the maximum number of hits for a read = 10. Each day’s transcripts were grouped as being housekeeping or secretory and according to functional class. Expression values (TPM > 0.5) were used to determine sex dependant expression patterns over time.

### Differential expression of housekeeping and secretory proteins

Quality filtered sequence reads for each time point were analysed against the de novo assembled transcriptome as reference using CLC Genomics Workbench (12.0). A principle component analysis as a function of time on the unclustered datasets determined the amount of variance in the transcriptomes of males and females during the feeding period using normalized log counts per million (CPM) values in CLC Genomics Workbench (12.0). This incorporated the use of the trimmed mean of M values (TMM) normalization method^110^ as used in EdgeR or DESeq2 packages. This method was also suggested for exploratory studies without biological replicates. In such a case, the algorithm shares data between genes with similar expression to estimate technical and biological variability.

To identify differentially expressed genes by male and female ticks across the feeding period a metadata table of male and female reads respectively was constructed to analyse the variance (ANOVA) across groups to test differential expressions due to day on CLC Genomics Workbench (12.0). A volcano plot was used as visual tool to demonstrate structure in the data and to display both the fold-change and statistical significance (the -log10 of the *p* value) while taking noise level into account when performing gene filtering. Differentially expressed transcripts (FDR value < 0.05) with a log2 fold change larger than 4 and smaller than − 4 were extracted.

## Supplementary Information

Supplementary Figures.

Supplementary Table S1.

## References

[1] Francischetti IM, Sa-Nunes A, Mans BJ, Santos IM, Ribeiro JM (2009). The role of saliva in tick feeding. Front. Biosci. (Landmark Ed.).

[2] Mans BJ, Featherston J, de Castro MH, Pienaar R (2017). Gene duplication and protein evolution in tick-host interactions. Front. Cell. Infect. Microbiol..

[3] Mans BJ (2008). Comparative sialomics between hard and soft ticks: implications for the evolution of blood-feeding behavior. Insect Biochem. Mol. Biol..

[4] Mans BJ (2020). Quantitative visions of reality at the tick-host interface: biochemistry, genomics, proteomics and transcriptomics as measures of complete inventories of the tick sialoverse. Front. Cell. Infect. Microbiol..

[5] Karim S, Ribeiro JM (2015). An Insight into the sialome of the Lone Star tick, *Amblyomma americanum*, with a glimpse on its time dependent gene expression. PLoS ONE.

[6] Kotsyfakis M, Schwarz A, Erhart J, Ribeiro JM (2015). Tissue- and time-dependent transcription in *Ixodes ricinus* salivary glands and midguts when blood feeding on the vertebrate host. Sci. Rep..

[7] Perner J, Kropáčková S, Kopáček P, Ribeiro JMC (2018). Sialome diversity of ticks revealed by RNAseq of single tick salivary glands. PLoS Negl. Trop. Dis..

[8] Chidozie E, Anyaegbunam LC, Emmy-Egbe IO (2018). Review of emerging and re-emerging animal parasitic diseases. COOU Interdiscip. Res. J..

[9] de Waal DT (1992). Equine piroplasmosis: a review. Br. Vet. J..

[10] Hamel HD, Gothe R (1978). Influence of infestation rate of tick-paralysis in sheep induced by *Rhipicephalus evertsi evertsi* Neumann, 1897. Vet. Parasitol..

[11] Stampa S (1959). Tick paralysis in the Karoo areas of South Africa. Onderstepoort J. Vet. Res..

[12] Pienaar R, Neitz AWH, Mans BJ (2018). Tick paralysis: solving an enigma. Vet. Sci..

[13] Li W, Godzik A (2006). Cd-hit: a fast program for clustering and comparing large sets of protein or nucleotide sequences. Bioinformatics (Oxford, England).

[14] Altschul SF, Gish W, Miller W, Myers EW, Lipman DJ (1990). Basic local alignment search tool. J. Mol. Biol..

[15] Simao FA, Waterhouse RM, Ioannidis P, Kriventseva EV, Zdobnov EM (2015). BUSCO: assessing genome assembly and annotation completeness with single-copy orthologs. Bioinformatics.

[16] de Castro MH (2016). De novo assembly and annotation of the salivary gland transcriptome of *Rhipicephalus appendiculatus* male and female ticks during blood feeding. Ticks Tick Borne Dis..

[17] Xavier MA (2019). Tick Gené's organ engagement in lipid metabolism revealed by a combined transcriptomic and proteomic approach. Ticks Tick Borne Dis..

[18] Tirloni L (2020). A physiologic overview of the organ-specific transcriptome of the cattle tick *Rhipicephalus microplus*. Sci. Rep..

[19] Tan AW, Francischetti IM, Slovak M, Kini RM, Ribeiro JM (2015). Sexual differences in the sialomes of the zebra tick, *Rhipicephalus pulchellus*. J. Proteom..

[20] Tirloni L (2020). Integrated analysis of sialotranscriptome and sialoproteome of the brown dog tick *Rhipicephalus sanguineus* (s.l.): insights into gene expression during blood feeding. J. Proteom..

[21] de Castro MH, de Klerk D, Pienaar R, Rees DJG, Mans BJ (2017). Sialotranscriptomics of *Rhipicephalus zambeziensis* reveals intricate expression profiles of secretory proteins and suggests tight temporal transcriptional regulation during blood-feeding. Parasit. Vectors.

[22] Cabezas-Cruz A, Espinosa P, Alberdi P, de la Fuente J (2019). Tick-pathogen interactions: the metabolic perspective. Trends Parasitol..

[23] Kyriakis JM, Avruch J (2012). Mammalian MAPK signal transduction pathways activated by stress and inflammation: a 10-year update. Physiol. Rev..

[24] Mans BJ (2019). Chemical equilibrium at the tick-host feeding interface: a critical examination of biological relevance in hematophagous behavior. Front. Physiol..

[25] Kelley LA, Mezulis S, Yates CM, Wass MN, Sternberg MJ (2015). The Phyre2 web portal for protein modeling, prediction and analysis. Nat. Protoc..

[26] Mans BJ, Ribeiro JM, Andersen JF (2008). Structure, function, and evolution of biogenic amine-binding proteins in soft ticks. J. Biol. Chem..

[27] Walker, J. B., Keirans, J. E. & Horak, I. G. The genus Rhipicephalus (Acari, Ixodidae): a guide to the Brown Ticks of the world. (Cambridge University Press, 2000).

[28] Mans BJ (2016). Ancestral reconstruction of tick lineages. Ticks Tick Borne Dis..

[29] Šimo L, Koči J, Žitňan D, Park Y (2011). Evidence for D1 dopamine receptor activation by a paracrine signal of dopamine in tick salivary glands. PLoS ONE.

[30] Šimo L, Koči J, Kim D, Park Y (2014). Invertebrate specific D1-like dopamine receptor in control of salivary glands in the black-legged tick *Ixodes scapularis*. J. Comp. Neurol..

[31] Kaufman WR, Phillips JE (1973). Ion and water balance in the ixodid tick *Dermacentor andersoni*. J. Exp. Biol..

[32] Šimo L, Koči J, Park Y (2013). Receptors for the neuropeptides, myoinhibitory peptide and SIFamide, in control of the salivary glands of the black-legged tick *Ixodes scapularis*. Insect Biochem. Mol. Biol..

[33] Sisto M, Ribatti D, Lisi S (2019). Aquaporin water channels: New perspectives on the potential role in inflammation. Adv. Protein Chem. Struct. Biol..

[34] Bullard R (2016). Structural characterization of tick cement cones collected from in vivo and artificial membrane blood-fed Lone Star ticks (*Amblyomma americanum*). Ticks Tick Borne Dis..

[35] Garcia GR (2014). The sialotranscriptome of *Amblyomma triste*, *Amblyomma parvum* and *Amblyomma cajennense* ticks, uncovered by 454-based RNA-seq. Parasite Vector.

[36] Hollmann T (2018). Identification and characterization of proteins in the *Amblyomma americanum* tick cement cone. Int. J. Parasitol..

[37] Wang H, Paesen GC, Nuttall PA, Barbour AG (1998). Male ticks help their mates to feed. Nature.

[38] Wang H, Nuttall PA (1995). Immunoglobulin-G binding proteins in the ixodid ticks, *Rhipicephalus appendiculatus Amblyomma variegatum* and *Ixodes hexagonus*. Parasitology.

[39] Wang H, Nuttall PA (1999). Immunoglobulin-binding proteins in ticks: New target for vaccine development against a blood-feeding parasite. Cell. Mol. Life Sci..

[40] Tisoncik JR (2012). Into the eye of the cytokine storm. Microbiol. Mol. Biol. Rev..

[41] Kotál J (2015). Modulation of host immunity by tick saliva. J. Proteom..

[42] Bhusal RP (2020). Evasins: tick salivary proteins that inhibit mammalian chemokines. Trends Biochem. Sci..

[43] Mans BJ (2011). Evolution of vertebrate hemostatic and inflammatory control mechanisms in blood-feeding arthropods. J. Innate Immun..

[44] Ribeiro JM (2012). The sialotranscriptome of *Antricola delacruzi* female ticks is compatible with non-hematophagous behavior and an alternative source of food. Insect Biochem. Mol. Biol..

[45] Tirloni L, Seixas A, Mulenga A, Vaz Ida S, Termignoni C (2014). A family of serine protease inhibitors (serpins) in the cattle tick *Rhipicephalus* (*Boophilus*) *microplus*. Exp. Parasitol..

[46] Francischetti IM, Mather TN, Ribeiro JM (2003). Cloning of a salivary gland metalloprotease and characterization of gelatinase and fibrin(ogen)lytic activities in the saliva of the Lyme disease tick vector *Ixodes scapularis*. Biochem. Biophys. Res. Commun..

[47] Francischetti IM, Mather TN, Ribeiro JM (2005). Tick saliva is a potent inhibitor of endothelial cell proliferation and angiogenesis. J. Thromb. Haemost..

[48] Šimo L, Kazimirova M, Richardson J, Bonnet SI (2017). The essential role of tick salivary glands and saliva in tick feeding and pathogen transmission. Front. Cell. Infect. Microbiol..

[49] Jarmey JM, Riding GA, Pearson RD, McKenna RV, Willadsen P (1995). Carboxydipeptidase from *Boophilus microplus*: a "concealed" antigen with similarity to angiotensin-converting enzyme. Insect Biochem. Mol. Biol..

[50] Ribeiro JM, Mather TN (1998). *Ixodes scapularis*: salivary kininase activity is a metallo dipeptidyl carboxypeptidase. Exp. Parasitol..

[51] Paesen GC, Adams PL, Harlos K, Nuttall PA, Stuart DI (1999). Tick histamine-binding proteins: isolation, cloning, and three-dimensional structure. Mol. Cell.

[52] Reichhardt MP (2020). An inhibitor of complement C5 provides structural insights into activation. Proc. Natl. Acad. Sci. USA.

[53] Jore MM (2016). Structural basis for therapeutic inhibition of complement C5. Nat. Struct. Mol. Biol..

[54] Lai R, Takeuchi H, Jonczy J, Rees HH, Turner PC (2004). A thrombin inhibitor from the ixodid tick, *Amblyomma hebraeum*. Gene.

[55] Koh CY (2007). Variegin, a novel fast and tight binding thrombin inhibitor from the tropical bont tick. J. Biol. Chem..

[56] Macedo-Ribeiro S (2008). Isolation, cloning and structural characterisation of boophilin, a multifunctional Kunitz-type proteinase inhibitor from the cattle tick. PLoS ONE.

[57] Zhu K (1997). Isolation and characterization of americanin, a specific inhibitor of thrombin, from the salivary glands of the lone star tick *Amblyomma americanum* (L.). Exp. Parasitol..

[58] Nienaber J, Gaspar AR, Neitz AW (1999). Savignin, a potent thrombin inhibitor isolated from the salivary glands of the tick *Ornithodoros savignyi* (Acari: Argasidae). Exp. Parasitol..

[59] van de Locht A (1996). The ornithodorin-thrombin crystal structure, a key to the TAP enigma?. EMBO J..

[60] Kim TK (2015). Conserved *Amblyomma americanum* tick Serpin19, an inhibitor of blood clotting factors Xa and XIa, trypsin and plasmin, has anti-haemostatic functions. Int. J. Parasitol..

[61] Rodriguez-Valle M, Xu T, Kurscheid S, Lew-Tabor AE (2015). *Rhipicephalus microplus* serine protease inhibitor family: annotation, expression and functional characterisation assessment. Parasites Vectors.

[62] Xu T, Lew-Tabor A, Rodriguez-Valle M (2016). Effective inhibition of thrombin by *Rhipicephalus microplus* serpin-15 (RmS-15) obtained in the yeast *Pichia pastoris*. Ticks Tick Borne Dis..

[63] Mans, B. J. In *Extracellular Composite Matrices in Arthropods* (eds E. Cohen & B. Moussian) Ch. Chapter 17, 625–688 (Springer International Publishing, 2016).

[64] Anisuzzaman (2011). Longistatin, a plasminogen activator, is key to the availability of blood-meals for ixodid ticks. PLoS Pathog..

[65] Francischetti IM (2005). The transcriptome of the salivary glands of the female western black-legged tick *Ixodes pacificus* (Acari: Ixodidae). Insect Biochem. Mol. Biol..

[66] Ribeiro JM (2006). An annotated catalog of salivary gland transcripts from *Ixodes scapularis* ticks. Insect Biochem. Mol. Biol..

[67] Batista IF (2010). A new Factor Xa inhibitor from *Amblyomma cajennense* with a unique domain composition. Arch. Biochem. Biophys..

[68] Chmelař J (2008). Insight into the sialome of the castor bean tick, *Ixodes ricinus*. BMC Genom..

[69] Rodriguez-Valle M (2018). Transcriptome and toxin family analysis of the paralysis tick, *Ixodes holocyclus*. Int. J. Parasitol..

[70] Vink S, Daly NL, Steen N, Craik DJ, Alewood PF (2014). Holocyclotoxin-1, a cystine knot toxin from *Ixodes holocyclus*. Toxicon.

[71] McNally KL (2012). Differential salivary gland transcript expression profile in *Ixodes scapularis* nymphs upon feeding or flavivirus infection. Ticks Tick Borne Dis..

[72] Ribeiro JMC, Francischetti IMB (2003). Role of arthropod saliva in blood feeding: sialome and post-sialome perspectives. Annu. Rev. Entomol..

[73] Fukumoto S, Sakaguchi T, You M, Xuan X, Fujisaki K (2006). Tick troponin I-like molecule is a potent inhibitor for angiogenesis. Microvasc. Res..

[74] You M (2001). Molecular characterization of a troponin I-like protein from the hard tick *Haemaphysalis longicornis*. Insect Biochem. Mol. Biol..

[75] Anatriello E (2010). An insight into the sialotranscriptome of the brown dog tick, *Rhipicephalus sanguineus*. BMC Genom..

[76] Esteves E (2017). Analysis of the salivary gland transcriptome of unfed and partially fed *Amblyomma sculptum* ticks and descriptive proteome of the saliva. Front. Cell. Infect. Microbiol..

[77] Karim S, Singh P, Ribeiro JMC, Oliveira PL (2011). A deep insight into the sialotranscriptome of the Gulf coast tick, *Amblyomma maculatum*. PLoS ONE.

[78] Ribeiro JM, Slovak M, Francischetti IM (2017). An insight into the sialome of *Hyalomma excavatum*. Ticks Tick Borne Dis..

[79] Miller, J. R. *et al.* A draft genome sequence for the *Ixodes scapularis* cell line, ISE6. *F1000Research* **7**, 297. 10.12688/f1000research.13635.1 (2018). 10.12688/f1000research.13635.1 PMC5883391 29707202

[80] Valenzuela JG (2002). Exploring the sialome of the tick *Ixodes scapularis*. J. Exp. Biol..

[81] Garcia GR (2020). A transcriptome and proteome of the tick *Rhipicephalus microplus* shaped by the genetic composition of its hosts and developmental stage. Sci. Rep..

[82] Mans, B. In *Biology of Ticks* Vol. 2 (ed D. E. Sonenshine, Roe, R. M.) Ch. 9, 220–239 (Oxford University Press, 2014).

[83] Kemp, D. H., Stone, B. F. & Binnington, K. C. Tick attachment and feeding: Role of the mouthparts, feeding apparatus, salivary gland secretions and the host response. *Physiology of Ticks*, 119–168 (1982).

[84] Schwarz A (2013). De novo *Ixodes ricinus* salivary gland transcriptome analysis using two next-generation sequencing methodologies. FASEB J..

[85] Mudenda L (2014). Proteomics informed by transcriptomics identifies novel secreted proteins in *Dermacentor andersoni* saliva. Int. J. Parasitol..

[86] Schwarz A (2014). A systems level analysis reveals transcriptomic and proteomic complexity in *Ixodes ricinus* midgut and salivary glands during early attachment and feeding. Mol. Cell. Proteom..

[87] Bensaoud C (2018). De novo assembly and annotation of *Hyalomma dromedarii* tick (Acari: Ixodidae) sialotranscriptome with regard to gender differences in gene expression. Parasites Vectors.

[88] Gong H (2014). Immunoglobulin G binding protein (IGBP) from *Rhipicephalus haemaphysaloides*: identification, expression, and binding specificity. Parasitol. Res..

[89] Sonenshine, D. E. & Coons, L. B. In *Biology of Ticks* Vol. 1 *Male reproductive system: anatomy, physiology, and molecular biology* (eds Daniel E. Sonenshine & R. Michael Roe) Ch. 18, 484–518 (Oxford University Press, Incorporated, 2013).

[90] Horáčková J, Rudenko N, Golovchenko M, Havlíková S, Grubhoffer L (2010). IrML—a gene encoding a new member of the ML protein family from the hard tick, *Ixodes ricinus*. J. Vector Ecol..

[91] Xiang FY, Zhou YZ, Zhou JL (2012). Identification of differentially expressed genes in the salivary gand of *Rhipicephalus haemaphysaloides* by the suppression subtractive hybridization approach. J. Integr. Agric..

[92] Shi H-J (2004). Cloning and characterization of rat spermatid protein SSP411: a thioredoxin-like protein. J. Androl..

[93] King KL, Stewart M, Roberts TM, Seavy M (1992). Structure and macromolecular assembly of two isoforms of the major sperm protein (MSP) from the amoeboid sperm of the nematode, *Ascaris suum*. J. Cell Sci..

[94] Heyne H, Elliott EG, Bezuidenhout JD (1987). Rearing and infection techniques for *Amblyomma* species to be used in heartwater transmission experiments. Onderstepoort J. Vet. Res..

[95] Chikhi R, Rizk G (2013). Space-efficient and exact de Bruijn graph representation based on a Bloom filter. Algorithms Mol. Biol..

[96] Grabherr MG (2011). Full-length transcriptome assembly from RNA-Seq data without a reference genome. Nat. Biotechnol..

[97] Senalik, D. *Find Open Reading Frames in a DNA or RNA Sequence*. https://github.com/vikas0633/perl/blob/master/orffinder.pl (2011).

[98] Giraldo-Calderon GI (2015). VectorBase: an updated bioinformatics resource for invertebrate vectors and other organisms related with human diseases. Nucleic Acids Res..

[99] Tatusov RL (2003). The COG database: an updated version includes eukaryotes. BMC Bioinform..

[100] Kanehisa M, Sato Y, Kawashima M, Furumichi M, Tanabe M (2016). KEGG as a reference resource for gene and protein annotation. Nucleic Acids Res..

[101] Kanehisa M, Sato Y, Morishima K (2016). BlastKOALA and GhostKOALA: KEGG tools for functional characterization of genome and metagenome sequences. J. Mol. Biol..

[102] Altschul SF (1997). Gapped BLAST and PSI-BLAST: a new generation of protein database search programs. Nucleic Acids Res..

[103] Petersen TN, Brunak S, von Heijne G, Nielsen H (2011). SignalP 4.0: discriminating signal peptides from transmembrane regions. Nat. Methods.

[104] Krogh A, Larsson B, von Heijne G, Sonnhammer EL (2001). Predicting transmembrane protein topology with a hidden Markov model: application to complete genomes. J. Mol. Biol..

[105] Kall L, Krogh A, Sonnhammer EL (2004). A combined transmembrane topology and signal peptide prediction method. J. Mol. Biol..

[106] Katoh K, Standley DM (2013). MAFFT multiple sequence alignment software version 7: Improvements in performance and usability. Mol. Biol. Evol..

[107] Minh BQ (2020). IQ-TREE 2: new models and efficient methods for phylogenetic iInference in the genomic era. Mol. Biol. Evol..

[108] Moreno-Hagelsieb G, Latimer K (2008). Choosing BLAST options for better detection of orthologs as reciprocal best hits. Bioinformatics (Oxford, England).

[109] Heberle H, Meirelles G, Telles G, da Silva F, Minghim R (2015). InteractiVenn: a web-based tool for the analysis of sets through Venn diagrams. BMC Bioinform..

[110] Robinson MD, Oshlack A (2010). A scaling normalization method for differential expression analysis of RNA-seq data. Genome Biol..

[111] Stutzer C, Mans BJ, Gaspar AR, Neitz AW, Maritz-Olivier C (2009). Ornithodoros savignyi: soft tick apyrase belongs to the 5'-nucleotidase family. Exp. Parasitol..

[112] Mulenga A, Kim T, Ibelli AM (2013). *Amblyomma americanum* tick saliva serine protease inhibitor 6 is a cross-class inhibitor of serine proteases and papain-like cysteine proteases that delays plasma clotting and inhibits platelet aggregation. Insect Mol. Biol..

[113] Tirloni L (2019). *Amblyomma americanum* serpin 27 (AAS27) is a tick salivary anti-inflammatory protein secreted into the host during feeding. PLoS Negl. Trop. Dis..

[114] Chmelař J (2011). A tick salivary protein targets cathepsin G and chymase and inhibits host inflammation and platelet aggregation. Blood.

[115] Gao X (2011). Characterization of the anticoagulant protein Rhipilin-1 from the *Rhipicephalus haemaphysaloides* tick. J. Insect Physiol..

[116] Cao J (2013). Characterization of a new Kunitz-type serine protease inhibitor from the hard tick *Rhipicephalus hemaphysaloides*. Arch. Insect Biochem. Physiol..

[117] Diaz-Martin V (2013). An insight into the proteome of the saliva of the argasid tick *Ornithodoros moubata* reveals important differences in saliva protein composition between the sexes. J. Proteom..

[118] Prevot PP (2006). Anti-hemostatic effects of a serpin from the saliva of the tick *Ixodes ricinus*. J. Biol. Chem..

[119] Déruaz M (2008). Ticks produce highly selective chemokine binding proteins with antiinflammatory activity. J. Exp. Med..

[120] Mulenga A, Macaluso KR, Simser JA, Azad AF (2003). The American dog tick, *Dermacentor variabilis*, encodes a functional histamine release factor homolog. Insect Biochem. Mol. Biol..

[121] Dai J (2010). Tick histamine release factor is critical for *Ixodes scapularis* engorgement and transmission of the lyme disease agent. PLoS Pathog.

[122] Singh K (2017). Yeast surface display identifies a family of evasins from ticks with novel polyvalent CC chemokine-binding activities. Sci. Rep..

