# Supplementary material for: De novo assembled salivary gland transcriptome and expression pattern analyses for Rhipicephalus evertsi evertsi Neuman, 1897 male and female ticks
Source: Sci Rep. 2021 Jan 15;11:1642. doi: 10.1038/s41598-020-80454-3 (PMC7810686; doi:10.1038/s41598-020-80454-3)
Supplement: Supplementary file 1 — Supplementary Figures. [file 41598_2020_80454_MOESM1_ESM.docx]

## *De novo* assembled salivary gland transcriptome and expression pattern analyses for *Rhipicephalus evertsi evertsi* Neuman, 1897 male and female ticks

## *Ronel Pienaar^1,2^, Daniel G. de Klerk^1^, Minique H. de Castro^3^, Jonathan Featherston^3^, *Ben J. Mans^1,2,4^

## ^1^Epidemiology, Parasites and Vectors, Agricultural Research Council-Onderstepoort Veterinary Research, Onderstepoort, South Africa, ^2^Department of Veterinary Tropical Diseases, University of Pretoria, Pretoria, South Africa, ^3^Agricultural Research Council-Biotechnology Platform, ^4^Department of Life and Consumer Sciences, University of South Africa, Pretoria, South Africa

## *Corresponding authors: [volschenkr@arc.agric.za](mailto:volschenkr@arc.agric.za); [mansb@arc.agric.za](mailto:mansb@arc.agric.za)


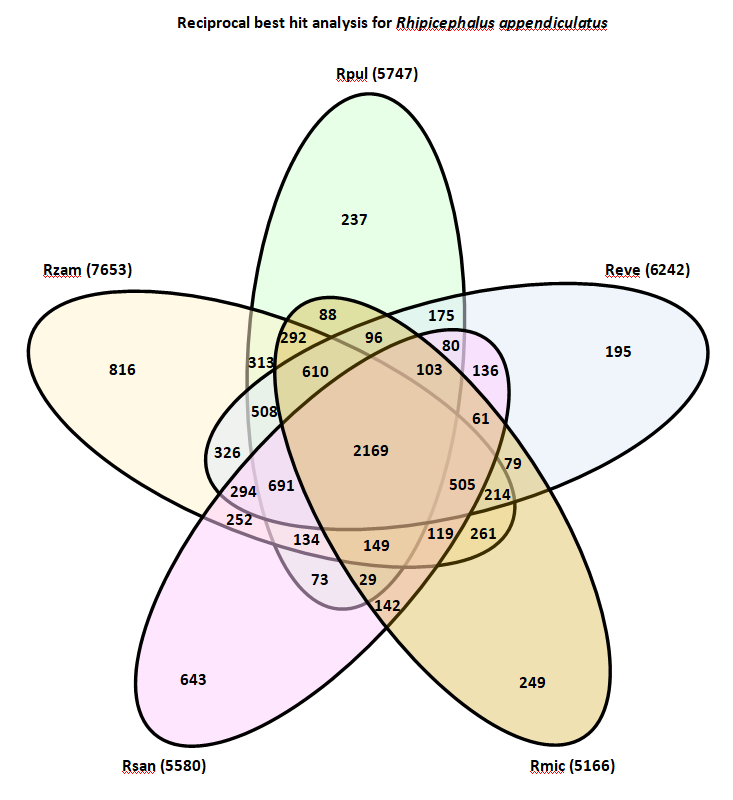


Figure 1: Venn diagram to show the number of shared orthologs found for the *Rhipicephalus appendiculatus* transcriptome based on reciprocal best hits with the other publically available transcriptomes for *Rhipicephalus microplus* (Rmic), *Rhipicephalus pulchellus* (Rpul), *Rhipicephalus sanguineus* (Rsan), *Rhipicephalus zambeziensis* (Rzam) and for *Rhipicephalus evertsi evertsi* (Reve) (this study). Numbers in parenthesis indicate total number of reciprocal best hits for each transcriptome and while those shared among transcriptomes are indicated in numbers.


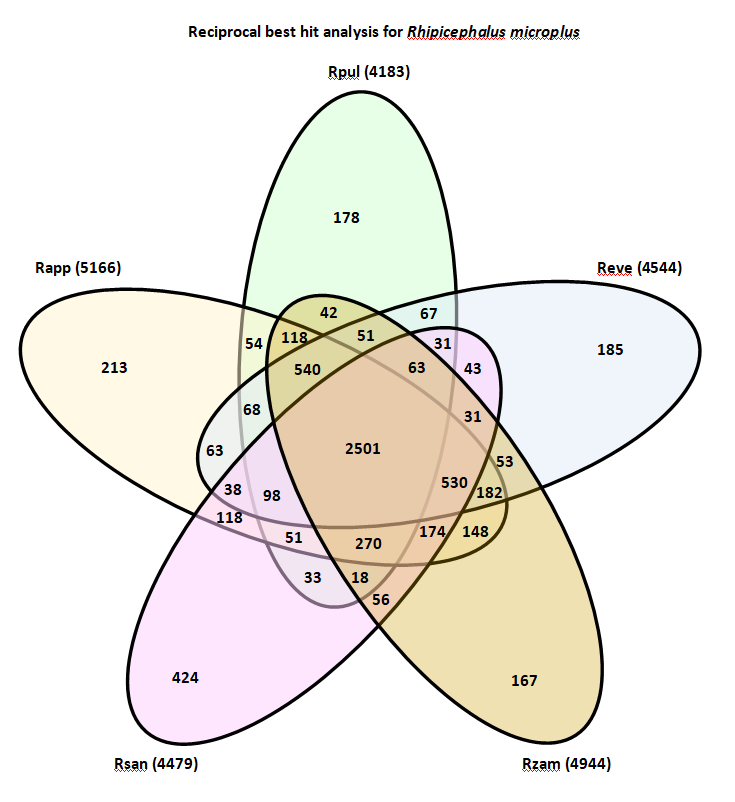


Figure 2: Venn diagram to show the number of shared orthologs found for the *Rhipicephalus microplus* transcriptome based on reciprocal best hits with the other publically available transcriptomes for *Rhipicephalus appendiculatus* (Rapp), *Rhipicephalus pulchellus* (Rpul), *Rhipicephalus sanguineus* (Rsan), *Rhipicephalus zambeziensis* (Rzam) and for *Rhipicephalus evertsi evertsi* (Reve) (this study). Numbers in parenthesis indicate total number of reciprocal best hits for each transcriptome and while those shared among transcriptomes are indicated in numbers.


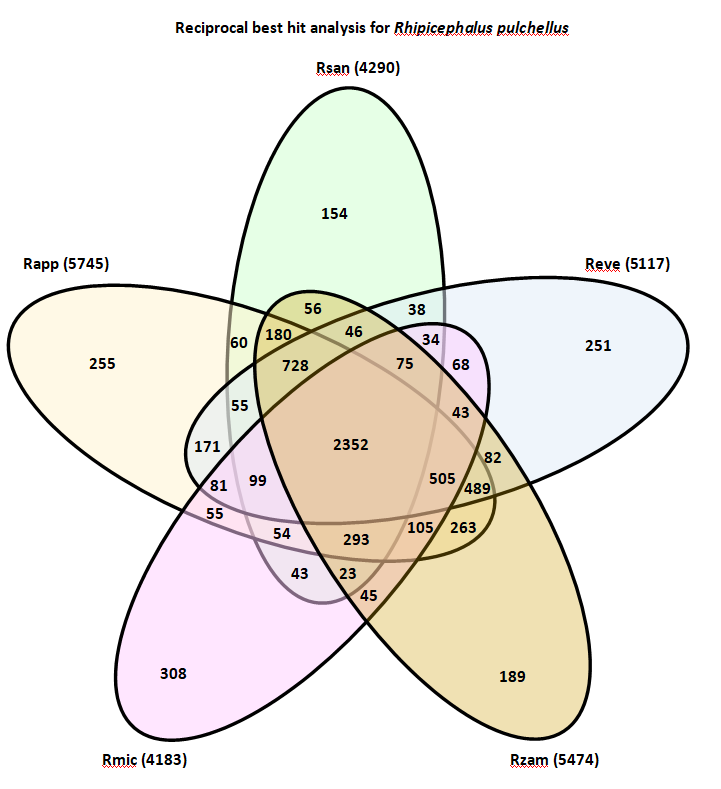


Figure 3: Venn diagram to show the number of shared orthologs found for the *Rhipicephalus pulchellus* transcriptome based on reciprocal best hits with the other publically available transcriptomes for *Rhipicephalus appendiculatus* (Rapp), *Rhipicephalus microplus* (Rmic), *Rhipicephalus sanguineus* (Rsan), *Rhipicephalus zambeziensis* (Rzam) and for *Rhipicephalus evertsi evertsi* (Reve) (this study). Numbers in parenthesis indicate total number of reciprocal best hits for each transcriptome and while those shared among transcriptomes are indicated in numbers.


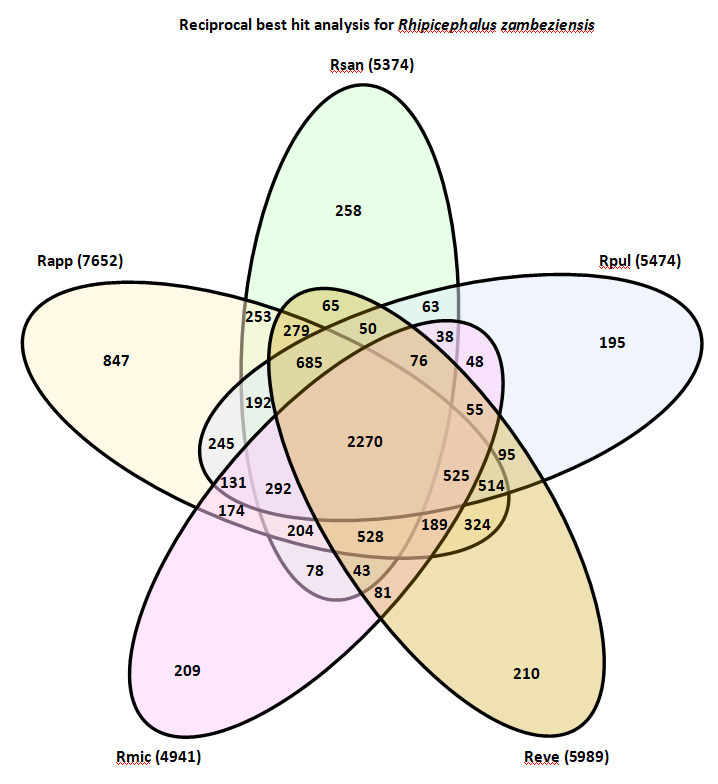


Figure 4: Venn diagram to show the number of shared orthologs found for the *Rhipicephalus zambeziensis* transcriptome based on reciprocal best hits with the other publically available transcriptomes for *Rhipicephalus appendiculatus* (Rapp), *Rhipicephalus microplus* (Rmic), *Rhipicephalus pulchellus* (Rpul), *Rhipicephalus sanguineus* (Rsan), and for *Rhipicephalus evertsi evertsi* (Reve) (this study). Numbers in parenthesis indicate total number of reciprocal best hits for each transcriptome and while those shared among transcriptomes are indicated in numbers.


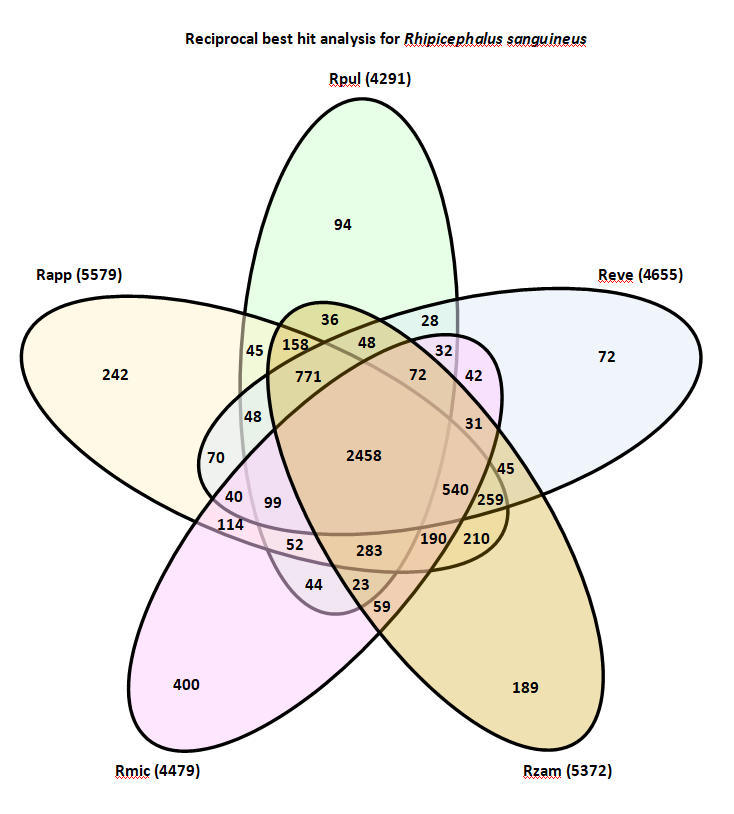


Figure 5: Venn diagram to show the number of shared orthologs found for the *Rhipicephalus sanguineus* transcriptome based on reciprocal best hits with the other publically available transcriptomes for *Rhipicephalus appendiculatus* (Rapp), *Rhipicephalus microplus* (Rmic), *Rhipicephalus pulchellus* (Rpul), *Rhipicephalus zambeziensis* (Rzam) and for *Rhipicephalus evertsi evertsi* (Reve) (this study). Numbers in parenthesis indicate total number of reciprocal best hits for each transcriptome and while those shared among transcriptomes are indicated in numbers.
