# Supplementary material for: De novo assembled salivary gland transcriptome and expression pattern analyses for Rhipicephalus evertsi evertsi Neuman, 1897 male and female ticks
Source: Sci Rep. 2021 Jan 15;11:1642. doi: 10.1038/s41598-020-80454-3 (PMC7810686; doi:10.1038/s41598-020-80454-3)
Supplement: Supplementary file 2 — Supplementary Table S1. [file 41598_2020_80454_MOESM2_ESM.docx]

**Table S1.** Statistics for quality trimming and creation of various data structures for the size set (450 bp - 1200 bp). Samples include female day 0 (F0), female day 2 (F2), female day 4 (F4), female day 6 (F6), all female samples combined (F), male day 0 (M0), male day 2 (M2), male day 4 (M4), male day 6 (M6), all male samples (M) and all samples combined (All). Data structures include raw reads before quality trimming (Untrimmed), paired reads that was quality trimmed and merged (Merged), Merged reads from which duplicates were removed (Mddup), single reads that was quality trimmed (Single), Single reads from which duplicates were removed (Sddup), combined Merged and Single reads (SM), combined Sddup and Mddup reads (SMddup). Also indicated are the average length in base pairs in parenthesis.

| *Samples* | Untrimmed  (AVL-bp) | Merged  (AVL-bp) | Mddup  (AVL-bp) | Single  (AVL-bp) | Sddup  (AVL-bp) | SM  (AVL-bp) | SMddup  (AVL-bp) |
| --- | --- | --- | --- | --- | --- | --- | --- |
| *F0* | 4,936,932  (300) | 982,911  (328) | 649,207  (329) | 3,045,180  (192) | 2,003,182  (230) | 4,028,091  (225) | 2,679,598  (232) |
| *F2* | 21,972,920  (300) | 1,326,260  (314) | 672,468  (317) | 4,834,431  (183) | 2,557,113  (234) | 6,160,691  (211) | 3,263,409  (182) |
| *F4* | 5,593,564  (300) | 941,826  (327) | 498,404  (331) | 3,034,374  (187) | 1,635,285 (239) | 3,976,200  (220) | 2,150,049  (195) |
| *F6* | 16,635,528  (300) | 1,144,529  (312) | 703,526  (314) | 3,772,746  (192) | 2,419,780  (232) | 4,917,275  (220) | 3,157,656  (223) |
| *F* | 49,138,944  (300) | 4,395,526  (320) | 2,285,916  (320) | 14,686,731  (188) | 7,908,359  (231) | 19,082,257  (219) | 10,271,244  (197) |
| *M0* | 47,640,252  (300) | 794,836  (290) | 539,702  (292) | 2,848,113  (152) | 1,914,185  (188) | 3,642,949  (182) | 2,478,604  (188) |
| *M2* | 13,163,944  (300) | 793,234  (281) | 475,328  (285) | 4,270,720  (140) | 2,489,530  (180) | 5,063,954  (162) | 2,986,860  (160) |
| *M4* | 13,374,500  (300) | 799,436  (277) | 474,463  (281) | 4,222,354  (141) | 2,397,065  (181) | 5,021,790  (163) | 2,896,242  (158) |
| *M6* | 10,921,828  (300) | 1,039,568  (297) | 557,934  (301) | 4,469,183  (149) | 2,399,357  (197) | 5,508,751  (177) | 2,983,957  (156) |
| *M* | 42,224,524  (300) | 3,427,074  (287) | 1,890,069  (285) | 15,810,370  (145) | 8,921,253  (171) | 19,237,444  (170) | 10,924,684  (159) |
| *All* | 91,363,468  (300) | 7,822,600  (305) | 4,734,355  (318) | 30,497,101  (166) | 16,984,596  (184) | 38,319,701  (194) | 21,953,443  (231) |
